# Reassessment of the Evidence for Postcranial Skeletal Pneumaticity in Triassic Archosaurs, and the Early Evolution of the Avian Respiratory System

**DOI:** 10.1371/journal.pone.0034094

**Published:** 2012-03-28

**Authors:** Richard J. Butler, Paul M. Barrett, David J. Gower

**Affiliations:** 1 GeoBio-Center, Ludwig-Maximilians-Universität München, Munich, Germany; 2 Department of Palaeontology, Natural History Museum, London, United Kingdom; 3 Department of Zoology, Natural History Museum, London, United Kingdom; Raymond M. Alf Museum of Paleontology, United States of America

## Abstract

Uniquely among extant vertebrates, birds possess complex respiratory systems characterised by the combination of small, rigid lungs, extensive pulmonary air sacs that possess diverticula that invade (pneumatise) the postcranial skeleton, unidirectional ventilation of the lungs, and efficient crosscurrent gas exchange. Crocodilians, the only other living archosaurs, also possess unidirectional lung ventilation, but lack true air sacs and postcranial skeletal pneumaticity (PSP). PSP can be used to infer the presence of avian-like pulmonary air sacs in several extinct archosaur clades (non-avian theropod dinosaurs, sauropod dinosaurs and pterosaurs). However, the evolution of respiratory systems in other archosaurs, especially in the lineage leading to crocodilians, is poorly documented. Here, we use µCT-scanning to investigate the vertebral anatomy of Triassic archosaur taxa, from both the avian and crocodilian lineages as well as non-archosaurian diapsid outgroups. Our results confirm previous suggestions that unambiguous evidence of PSP (presence of internal pneumatic cavities linked to the exterior by foramina) is found only in bird-line (ornithodiran) archosaurs. We propose that pulmonary air sacs were present in the common ancestor of Ornithodira and may have been subsequently lost or reduced in some members of the clade (notably in ornithischian dinosaurs). The development of these avian-like respiratory features might have been linked to inferred increases in activity levels among ornithodirans. By contrast, no crocodile-line archosaur (pseudosuchian) exhibits evidence for unambiguous PSP, but many of these taxa possess the complex array of vertebral laminae and fossae that always accompany the presence of air sacs in ornithodirans. These laminae and fossae are likely homologous with those in ornithodirans, which suggests the need for further investigation of the hypothesis that a reduced, or non-invasive, system of pulmonary air sacs may be have been present in these taxa (and secondarily lost in extant crocodilians) and was potentially primitive for Archosauria as a whole.

## Introduction

Birds are the most speciose extant terrestrial vertebrates, and their success has frequently been suggested to be associated with high metabolic rates and flight. Linked to these key innovations is the presence of an extensive system of air sacs in the thorax and abdomen, which form important components of the exceptionally efficient avian respiratory system [1]–[4]. The air sacs of birds reflect the near complete separation of the respiratory system into pump (the air sacs, in which gas exchange does not occur) and exchanger (the neopulmo and palaeopulmo) [2]–[4]. Finger-like extensions of the air sacs (pneumatic diverticula), as well as extensions of other components of the respiratory system, penetrate and pneumatize the axial and appendicular skeletons in most volant birds [1]–[3], [5]–[8]. Reduction of skeletal mass has often been cited as a key outcome of skeletal pneumatisation [1], [7]–[13], although recent work has suggested that avian bones are highly dense and therefore not necessarily ‘lightweight’ in absolute terms, but are light relative to their strength [14].

Other extant tetrapods including crocodilians (the closest living relatives of birds and the only other extant group of archosaurs) lack postcranial pneumatization and air sacs [7], [15], although crocodilians (and other sauropsids) possess sac-like chambers with a low density of parenchyma (the gas exchange tissue) [3], [7], [16]–[18] that are analogous to true (non-exchange) air sacs, which provide a foundation for the evolution of pneumatisation [7], and which have been inferred to have been present in the ancestral archosaur [18]. Evidence for postcranial skeletal pneumaticity (PSP) has been recognised in several extinct Mesozoic groups among bird-line archosaurs (Ornithodira), including non-crown-group Mesozoic birds such as *Archaeopteryx* and *Jeholornis* [7], [19]–[21], non-avian theropod [6], [7], [12], [15], [22]–[24] and sauropodomorph [9], [10], [15], [18], [22], [25]–[31] dinosaurs, and pterosaurs [7], [11], [15], [32], [33]. PSP has been used as a key source of evidence in investigations of the early evolution of the avian air sac system, with cervical and abdominal air sacs and an avian-style aspiration pump inferred to have been present in theropod dinosaurs and pterosaurs [6], [11]. These inferences have been made largely based on the observation that particular regions of the vertebral column are invariably pneumatised by particular air sacs in extant birds ([6], contra [24]). Air sacs are also hypothesised to have been present in sauropods [26], [27], [29], [30], although their function is less well established [6], [18]. However, in spite of the high level of interest in PSP and the evolution of the avian lung, the timing of the origin(s) of PSP in archosaurs is not well constrained, and the distribution of pneumaticity among early archosaurs and closely related taxa (Archosauriformes) remains controversial and poorly known.

**Figure 1:**
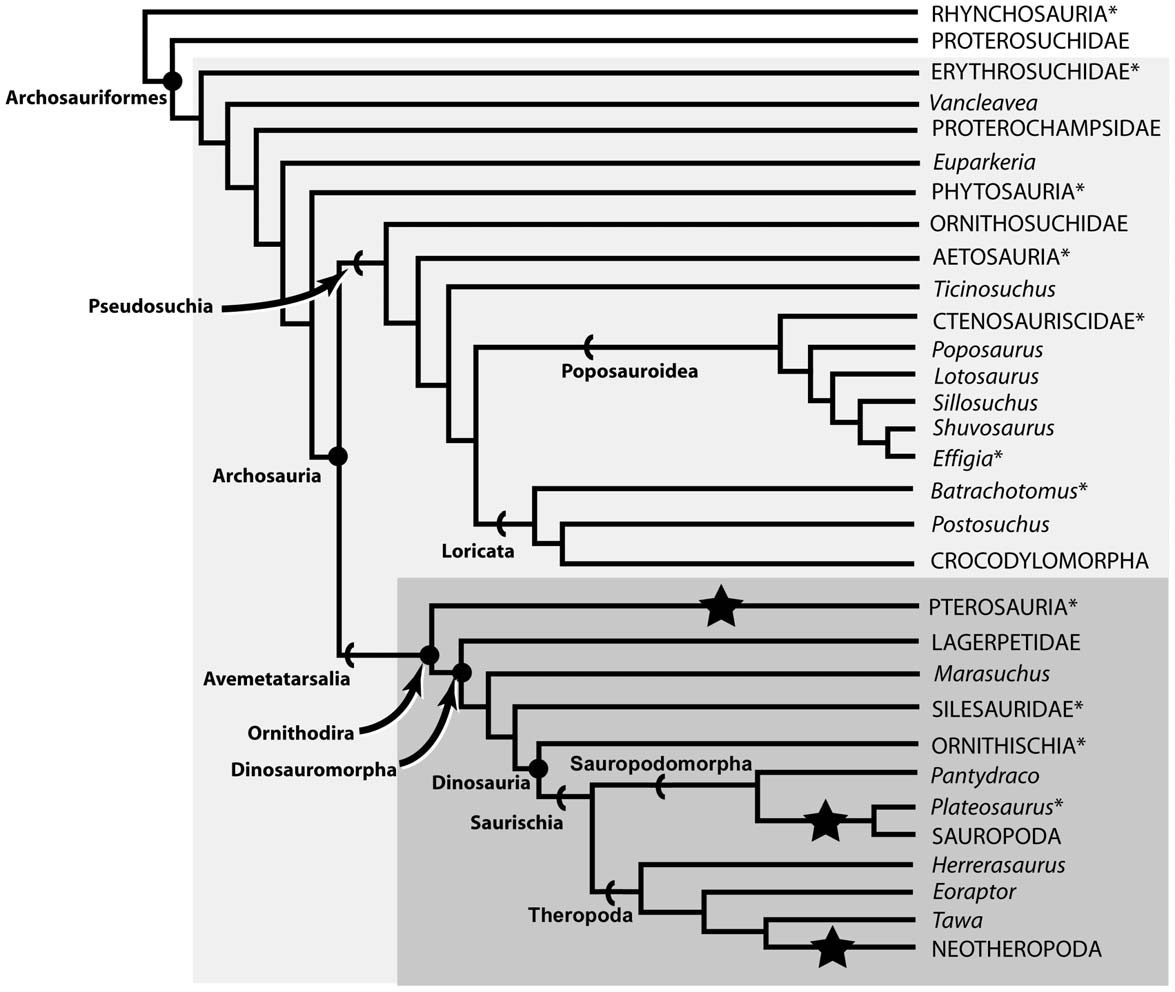
Simplified overview of Triassic archosauriform phylogeny based upon Nesbitt [**48**] showing relationships of major clades. Taxa marked with an asterisk were sampled for micro-CT scanning as part of this study. Stars indicate clades with unambiguous osteological evidence for postcranial skeletal pneumaticity (pterosaurs, neotheropods, most sauropodomorphs). The dark grey box delimits the clade (Ornithodira) for which we propose a bird-like air sac system was present. The light grey box delimits the minimum clade for which Gower [34] suggested postcranial skeletal pneumaticity might be present.

Gower [34] documented the presence of vertebral laminae, fossae, and associated foramina in several early archosauriforms, focusing primarily on the non-archosaurian archosauriform *Erythrosuchus africanus* from the early Middle Triassic of South Africa. Similar features on the external surfaces of the vertebrae in birds, non-avian saurischian (Theropoda + Sauropodomorpha) dinosaurs, and pterosaurs have often been interpreted as evidence of PSP (e.g. [15], [22]); on this basis, Gower [34] suggested that PSP might have been present in non-archosaurian archosauriforms (Fig. 1), so that some fundamental components of an avian-like lung (such as anteriorly and posteriorly positioned air sacs) may have been present in the last common ancestor of birds and crocodilians. O'Connor [7] subsequently re-examined axial material of *E. africanus* as well as material of phytosaurs (considered members of either the crocodilian stem group or as non-archosaurian archosauriforms, see below), and concluded that these taxa lacked unambiguous evidence for PSP, and that the features described by Gower [34] were likely vascular in origin (see also: [29]). However, it remains the case that features similar to those documented by Gower [34] have been and still are used to infer possible PSP in a wider range of Triassic archosauriforms (e.g. [25], [33]–[38]). Additionally, assessment of the presence/absence of PSP in Triassic archosauriforms has largely been based upon examination of external morphology (as well as limited examination of broken surfaces: [7], [34]). Moreover, the presence/absence of PSP has yet to be assessed in detail for a wide range of other extinct archosaur and archosauriform taxa. As a result, confusion remains as to the true distribution of PSP among major archosauriform lineages and its possible homology. For example, Nesbitt and Norell ([39]:1047) noted the presence of “true pleurocoels” on the anterior cervical vertebrae of the crocodilian-line archosaur *Effigia okeeffeae* from the Late Triassic of the USA; this statement has subsequently been cited as evidence of PSP in this taxon [24], [40], but the possible homology with avian PSP and its far-reaching implications have not been addressed.

Here, we survey the evidence for the presence/absence of PSP in a broad range of Triassic archosauriform and archosaur taxa, based upon first-hand examination of specimens, a review of the literature, comparative data on internal vertebral anatomy of extant sauropsids (both pneumatic and non-pneumatic taxa), and detailed examination of the internal structure of fossil vertebrae using micro-computed tomography (µCT). We focus in particular upon the previously neglected pseudosuchian lineage as well as previously understudied ornithodirans (e.g. ornithischians, *Silesaurus*). Finally, we synthesise our results with previous work and attempt to constrain the distribution and evolution of this PSP among early archosaurs.

### Overview of the phylogeny of early archosaurs

The phylogeny of early archosauriforms and archosaurs is an area of active study and considerable controversy [41]–[48], with the relationships among early crocodilian-line archosaurs (Pseudosuchia, also referred to as Crurotarsi by many authors, although see [48]) particularly contentious. Current views on archosaurian phylogeny are summarised in Figure 1 and are based primarily upon Nesbitt [48]. In the taxonomy used here, Archosauria refers to the crown clade consisting of crocodilians, birds, their common ancestor and all of its descendents ([49]; though see Benton [44], [45] for an alternative view). Archosauriformes refers to the clade consisting of archosaurs, *Proterosuchus*, their common ancestor and all of its descendents [50]. Non-archosaurian archosauriforms include proterosuchids, erythrosuchids, *Vancleavea*, *Euparkeria*, proterochampsids, doswelliids and possibly phytosaurs (e.g. [42]–[44], [48], [51], [52]). The more inclusive taxon Archosauromorpha includes all taxa more closely related to archosaurs than lepidosauromorphs, including predominantly Triassic forms such as trilophosaurids, ‘Protorosauria’ and rhynchosaurs in addition to archosauriforms. Although this study focuses primarily on archosauriforms, non-archosauriform archosauromorphs will be considered briefly, because they form a series of outgroups to archosauriforms and because one archosauromorph group (Rhynchosauria) was mentioned in the context of PSP by Gower [34].

Bird-line archosaurs (Avemetatarsalia) include dinosaurs, a number of non-dinosaurian dinosauromorph taxa such as *Marasuchus*, and probably pterosaurs. The clade including pterosaurs and dinosauromorphs is termed Ornithodira [41], and in terms of taxonomic content is identical to Avemetatarsalia at present. The inclusion of pterosaurs within Avemetatarsalia [41]–[45], [47], [48] is slightly controversial, and they have also been positioned phylogenetically close to ‘prolacertiform’ archosauromorphs by some analyses [53]–[55], although this is currently a minority view. The general scheme of relationships between other early ornithodirans and early dinosaurs is relatively uncontroversial [41], [44], [45], [48], [56]–[58] with a few exceptions: *Silesaurus* has been considered as a possible early ornithischian dinosaur [56], [59], although published phylogenetic analyses place it firmly within a silesaurid clade outside of Dinosauria [48], [56]–[58]; herrerasaurids (*Herrerasaurus*, *Staurikosaurus*) and *Eoraptor* have been considered early theropod dinosaurs [38], [48], [60]–[62], although some phylogenetic analyses place them as saurischians outside of the Theropoda/Sauropodomorpha split [56], [57] or place *Eoraptor* as a non-sauropod sauropodomorph [63]. Within Dinosauria the monophyly of Ornithischia and Saurischia are uncontroversial at present.

Pseudosuchia includes ornithosuchids, aetosaurs, crocodylomorphs, and an assemblage of ‘rauisuchian’ taxa. The exact nature of the probable para/polyphyly of this latter group is uncertain (e.g. [48], [64], [65]), but there is increasing evidence for a monophyletic Poposauroidea that includes ctenosauriscids [47], [48], [66]. Phytosaurs have generally been included within Pseudosuchia (e.g. [41], [47]), but new work suggests that they may instead be placed outside of Archosauria, as non-archosaurian archosauriforms [48]. Relationships among pseudosuchians generally and among ‘rauisuchians’ are highly unstable with little agreement between alternative phylogenetic hypotheses [41]–[45], [47], [48]. We here use the phylogeny of Nesbitt [48] as the primary framework for our discussion, because it is the most detailed analysis of Triassic archosaur interrelationships yet conducted.

The earliest archosauriforms originated in the Permian [67], but the vast majority of non-archosaurian archosauriform, early pseudosuchian, and early ornithodiran lineages are Triassic in age, with the radiation of crown group archosaurs likely beginning in the Early Triassic (252.3–247.2 Ma: [68]) or possibly the late Permian [48], [66], [67].

### Anatomical nomenclature

**Figure 2:**
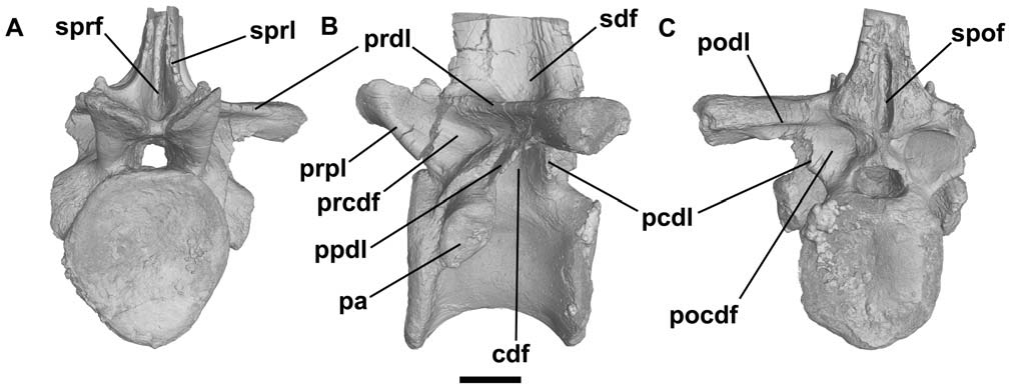
Holotype of the ctenosauriscid poposauroid *Hypselorhachis mirabilis* (NHMUK R16586, dorsal vertebra; with the elongate neural spine removed). Anterior (A), left lateral (B) and posterior (C) views, illustrating many of the typical vertebral laminae and fossae present in Triassic archosauriform vertebrae. Abbreviations: cdf, centrodiapophyseal fossa; pa, parapophysis; pcdl, posterior centrodiapophyseal lamina; pocdf, postzygapophyseal centrodiapophyseal fossa; podl, postzygodiapophyseal lamina; ppdl, paradiapophyseal lamina; prcdf, prezygapophyseal centrodiapophyseal fossa; prdl, prezygodiapophyseal lamina; prpl, prezygaparapophyseal lamina; sdf, spinodiapophyseal fossa; spof, spinopostzygapophyseal fossa; sprf, spinosprezygapophyseal fossa; sprl, spinoprezygapophyseal lamina. After Butler et al. (2009b). Scale bar equals 10 mm.

We follow the terminology and associated abbreviations for vertebral laminae outlined by Wilson [25], which named laminae based upon the basis of the homologous structures that they connect, and the terminology for vertebral fossae proposed by Wilson et al. [69] (see Figure 2). Abbreviations: ACDL, anterior centrodiapophyseal lamina; ACPL, anterior centroparapophyseal lamina; CPOL, centropostzygapophyseal lamina; CPRL, centroprezygapophyseal lamina; PCDL, posterior centrodiapophyseal lamina; PPDL, paradiapophyseal lamina; PODL, postzygadiapophyseal lamina; PRDL, prezygadiapophyseal lamina; PRPL, prezygaparapophyseal lamina; SPOL, spinopostzygapophyseal lamina; SPRL, spinoprezygapophyseal lamina; TPOL, infrapostzygapophyseal lamina; TPRL, infraprezygapophyseal lamina.

### Osteological correlates of pneumaticity and the recognition of PSP in fossil archosauromorphs

Britt's unpublished PhD thesis [15] was the first study to extensively review patterns of PSP in non-avian dinosaurs and pterosaurs. Based upon examination of osteological material of the extant birds *Struthio* and *Dromaius*, Britt ([15]:56) identified a number of characters that he suggested could be used to identify pneumatic bones in the fossil record, including large external foramina, external fossae with ‘crenulated texture’, well-developed neural arch laminae, thin outer walls, broad smooth or crenulated pneumatic tracks, and internal chambers connected to the exterior of the element by foramina. These features have subsequently been used to identify PSP in fossil material (e.g. [19], [20], [24], [29], [33], [35]). O'Connor [7] provided an extensive review of PSP in archosauriforms and re-evaluated previously proposed indicators (osteological correlates) of PSP: he recognised that many of these features, particularly foramina, (at least shallow) fossae, and neural arch laminae, are present to some degree in extant crocodilians, which lack PSP. As a result, the presence of such features in fossil taxa might indicate pneumaticity, but could alternatively indicate the influence of some other soft tissue system on the form of bones. Thus, these features cannot be considered as *unambiguous* evidence of PSP. O'Connor ([7]:fig. 12) defined a “pneumaticity profile”, indicating the correlation between osteological features and different soft-tissue systems. External fossae may result from muscle attachment, fat deposits, or outgrowths of the lungs, while external foramina indicate vasculature or pneumatic diverticula. Only the presence of large internal cavities/chambers that are connected to the exterior of the bone by large pneumatic cortical bone foramina or fossae can be considered unambiguous evidence of PSP [7], [22]. Note that bones that contain internal chambers but lack such a connection to the exterior are not pneumatic, but were likely filled with marrow or fatty tissues in life.

Here, we follow the criteria of O'Connor [7] for recognising unambiguous evidence of PSP. However, we also document the presence and distribution of other features (particularly fossae, foramina, and laminae) that provide ambiguous, but potentially important, evidence of possible PSP.

### Institutional abbreviations

AMNH, American Museum of Natural History, New York, USA; BIRUG, Lapworth Museum of Geology, University of Birmingham, Birmingham, UK; CAMZM, University Museum of Zoology, University of Cambridge, Cambridge, UK; NHMUK OR, NHMUK R, or NHMUK RU, Department of Palaeontology, Natural History Museum, London, UK; NHMUK RR, extant reptilian collections, Natural History Museum, London, UK; PVL, Fundación Miguel Lillo, Universidad Nacional de Tucumán, San Miguel de Tucumán, Argentina; PVSJ, Museo de Ciencias Naturales, Universidad Nacional de San Juan, Argentina; SMNS, Staatliches Museum für Naturkunde, Stuttgart, Germany; ZPAL, Institute of Paleobiology, Polish Academy of Sciences, Warsaw, Poland.

## Results

### Comparative CT-data for extant sauropsid taxa

#### *Varanus komodoensis*, Lepidosauria

NHMUK RR 1934.9.2.2, three dorsal vertebrae.

**Figure 3:**
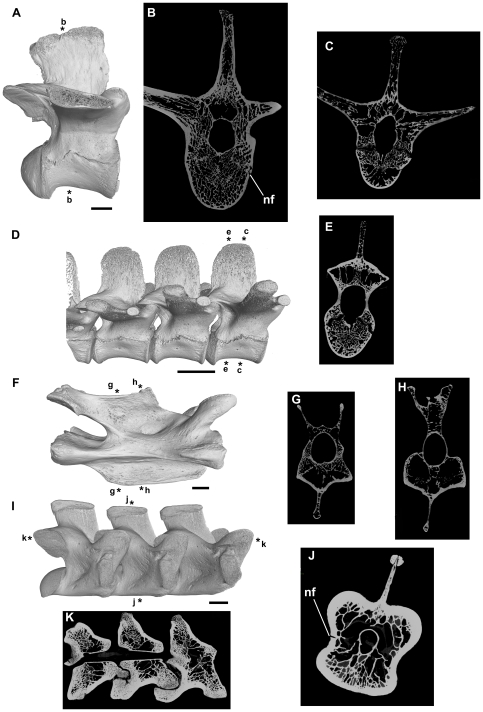
Vertebrae of extant reptiles lacking postcranial skeletal pneumaticity. A, B: *Alligator mississippiensis*, NHMUK RR 73.2.21.2, right lateral view (A) and transverse section (B). C–E: *Alligator mississippiensis*, NHMUK RR 1975.1423, transverse sections (C, E) and right lateral view (D). F–H: *Chelonoidis nigra abingdoni*, NHMUK RR 76.6.21.44, right lateral view (F), and transverse sections (G, H). I–K, *Varanus komodoensis*, NHMUK RR 1934.9.2.2, right lateral view (I, rendering of CT data) and transverse (J) and axial (K) sections. Asterisks adjacent to renderings indicate positions of sections. Abbreviation: nf, nutrient foramina. Scale bars equal 10 mm.

NHMUK RR 1934.9.2.2 is a series of three articulated dorsal vertebrae (Fig. 3I–K). Sections show a thick external layer of cortical bone and a small neural canal (Fig. 3K). There is a high degree of heterogeneity in the density of internal trabeculae. At the approximate midpoint of centrum length there are very large, interconnected spaces positioned mostly lateral and dorsal to the neural canal (Fig. 3J, K). Remnants of unidentified soft tissue appear to be present within most of these spaces. Very small (approximately 0.5 mm in diameter) foramina, presumably nutrient in origin, pierce the external surfaces of the centrum and arch and occasionally connect to these large internal cavities (Fig. 3J, K). In some cases these cavities have maximum dimensions that are more than 50% of the total height of the centrum and neural arch (Fig. 3K). By contrast, cancellous bone that is relatively more densely packed is positioned close to the ventral margin of the centrum and at its anterior and posterior ends (Fig. 3J, K). The most densely packed areas of bone lie lateral to the neural canal at the base of the postzygapophyses, within the anterior cotyles and posterior condyles of the centra and the articular surfaces of the pre- and postzygapophyses. Thus, a species that lacks pneumatisation shows features in vertebral cross sections that are reminiscent of structures (large intertrabecular spaces) that have sometimes been identified as evidence of PSP in fossil taxa. However, these large intertrabecular spaces do not connect to the exterior of the bone via large foramina and so are non-pneumatic in origin.

#### *Chelonoidis nigra abingdoni*, Testudines

NHMUK RR 76.6.21.44, single cervical vertebra.

NHMUK RR 76.6.21.44 is a cervical vertebra with an extensive median ventral keel, biconcave anterior and posterior articular facets, and concave depressions on the lateral surface of the arch at the base of the neural spine (Fig. 3F–H). The vertebra is very lightly constructed. In cross-section at the mid-point of centrum length, the vertebra is mostly hollow, with a large oval neural canal, large paired lateral spaces and a smaller median space in the centrum, and spaces within the neural arch dorsal to the neural canal (Fig. 3H). Towards each end of the vertebra there is a greater development of dense cancellous bone (Fig. 3G). The spaces in the centrum are incompletely separated from one another: moreover, they are traversed by sparsely distributed thin trabeculae. The external layer of cortical bone is often very thin (as little as 0.3 mm). There are no clear connections between the outside and the spaces within the centrum, with the exception of very small external foramina and associated narrow canals that extend through the cortical bone layer. Therefore, as in *Varanus komodoensis*, features are visible in vertebral cross sections that are reminiscent of structures (large intertrabecular spaces) that have sometimes been identified as evidence of PSP in fossil taxa. However, these features appear non-pneumatic in origin.

#### *Alligator mississippiensis*, Crocodilia

NHMUK RR 73.2.21.2, NHMUK RR 1975.1423, four dorsal vertebrae.

NHMUK RR 73.2.21.2 and NHMUK RR 1975.1423 lack fossae on the external surface of the centra/neural arches (Fig. 3A–E): however, small foramina are present over much of the neural arches and centra, and are especially abundant in shallow depressions at the base of the neural spines. CT cross-sections show there to be a relatively thick layer of dense external cortical bone, interior to which is relatively dense cancellous bone. The density of this cancellous bone is highly heterogeneous: the densest areas are at the base of the neural spine, the neurocentral suture, and the anterior and posterior ends of the centrum. By contrast, there are relatively large intertrabecular spaces above the neural canal and in the bases of the transverse processes. A particularly large vacuity (equal in transverse width to the neural canal) is visible at the posterior end of the vertebra. The small nutrient foramina that pierce the external walls generally extend through the external cortical bone and into the cancellous bone as narrow channels that maintain diameters equal to those of the external foramina.

#### *Struthio camelus*, Aves

NHMUK unnumbered (Department of Palaeontology osteological collection), first rib-bearing vertebra (cervical/thoracic junction).

**Figure 4:**
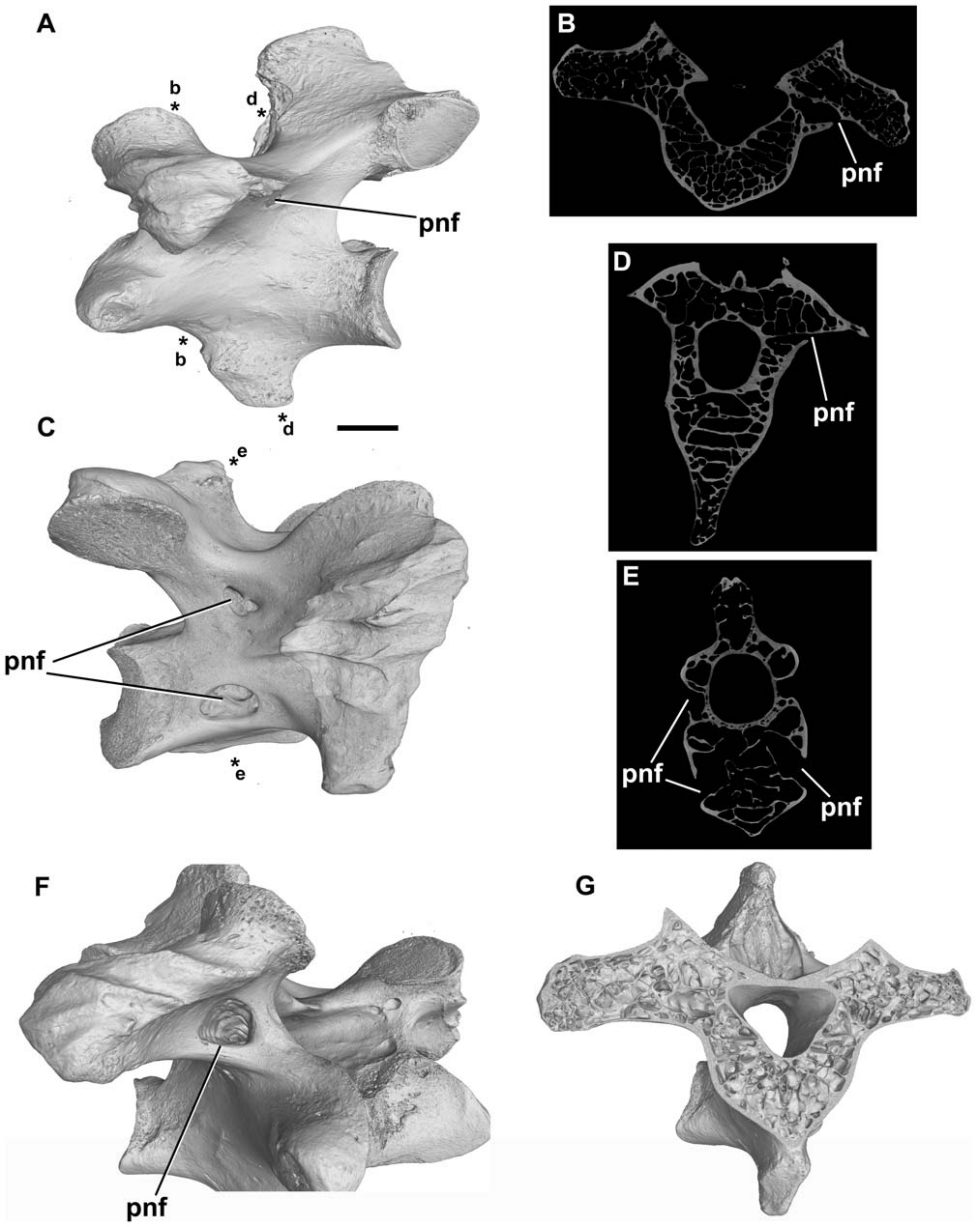
Ostrich, *Struthio camelus* (NHMUK unnumbered, first rib-bearing vertebra). Postcranial skeletal pneumaticity in an extant taxon. A, C: left (A) and right (C) lateral views. Asterisks mark the point of the cross-sections shown in B, D, and E. B, D, E: transverse sections through the vertebra. F: oblique right anterolateral view. G: cutaway of rendered model showing internal pneumatic cavities. Abbreviation: pnf, pneumatic foramen. Scale bar in A and C equals 10 mm.

This vertebra bears several foramina on its external surface (Fig. 4). There is a large opening on the anterior surface of the base of the right prezygapophysis that is presumably pneumatic in origin, while a cluster of much smaller foramina is present on the left side in the same position. Another small, presumably pneumatic, opening is visible on each side of the vertebra, at approximately midlength, lateral to the dorsal half of the neural canal. In cross-section there is a fairly thick external cortical bone layer that is comparable in proportional thickness to that observed in some of the crocodilian specimens. Nearly the entire vertebra, including the transverse processes and centrum, is composed of large interconnected chambers (air-filled in life), separated from one another by thin trabeculae. Areas of denser bone are limited to the anterior- and posteriormost ends of the centrum. The lateral foramina open into relatively small chambers that are, nonetheless, larger in diameter than the foramina that open into them. These chambers are connected to the other surrounding chambers. Likewise, the anterior foramina connect to the large internal chambers.

### Extinct non-Archosauromorpha

As discussed by Charig & Sues ([70]:17) and Benson et al. [12], many ‘pelycosaur’ synapsids (stem-mammals) possess deep fossae on the dorsolateral surfaces of precaudal neural arches ([71], [72]:fig. 8, 9), and there may be some development of a lamina on the neural arch, extending anteroventral from the diapophysis [12]. These features are superficially reminiscent of some of the vertebral fossae and pneumatic foramina of *Erythrosuchus* and many archosaurs, although not truly comparable to the very deep fossae and extremely well-developed laminae described below for many taxa. It is highly unlikely that the neural arch fossae and lamina of ‘pelycosaurs’ are the result of pneumatisation given their phylogenetic and stratigraphic distance from unambiguously pneumatic taxa [12].

### Extinct (mostly Triassic) Archosauromorpha

#### Archosauromorpha: Rhynchosauria

NHMUK R36618, cervical and dorsal vertebrae (*Stenaulorhynchus*, Middle Triassic, Tanzania). References: Benton [73], [74], Dilkes [75].

**Figure 5:**
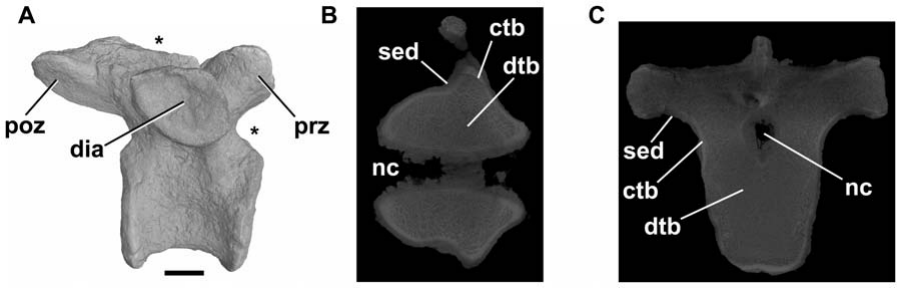
Rhynchosaur *Stenaulorhynchus stockleyi*, dorsal vertebra (NHMUK R36618). A: right lateral view. The asterisk positioned adjacent to the anterior margin shows the approximate position of section shown in B, whereas the asterisk positioned along the dorsal margin of the element corresponds to the approximate position of the transverse section shown in C. B, C: sections through the element. Abbreviations: ctb, cortical bone; dia, diapophysis; dtb, dense trabecular bone; nc, neural canal; poz, postzygapophysis; prz, prezygapophysis; sed, sediment. Scale bar equals 10 mm.

Gower ([34]:121) noted that pits, described as “deep pockets” ([75]:675) were present at the bases of the neural spines of the posterior dorsal and sacral vertebrae in the rhynchosaur *Howesia browni*; these pits are almost identical in position, size and morphology to those seen in ‘pelycosaur’ synapsids (see above). In the rhynchosaur *Stenaulorhynchus* (NHMUK R36618) the lateral surfaces of the centra are gently waisted (a feature common among tetrapods: see [29]) and there are very shallow depressions beneath the transverse processes in the cervical and dorsal vertebrae. However, vertebral laminae, distinct fossae, foramina, and other possible indicators of pneumaticity are absent (Fig. 5). CT sections (Fig. 5) show that the interior is composed of densely packed trabecular bone with no large spaces. Evidence of pneumaticity is therefore absent in *Stenaulorhynchus*, and potentially pneumatic features have not been reported in other rhynchosaurs [73], [74]. The “deep pockets” present on the vertebrae of *Howesia* therefore appear to be unique among rhynchosaurs, and were considered diagnostic for this taxon by Dilkes [75]. Given their positional and morphological similarity to features of ‘pelycosaur’ synapsids, it seems unlikely that these features are of pneumatic origin.

#### Archosauriformes: Proterosuchidae

References: Young [76], [77]; Cruickshank [78], Charig and Sues [70].

**Figure 6:**
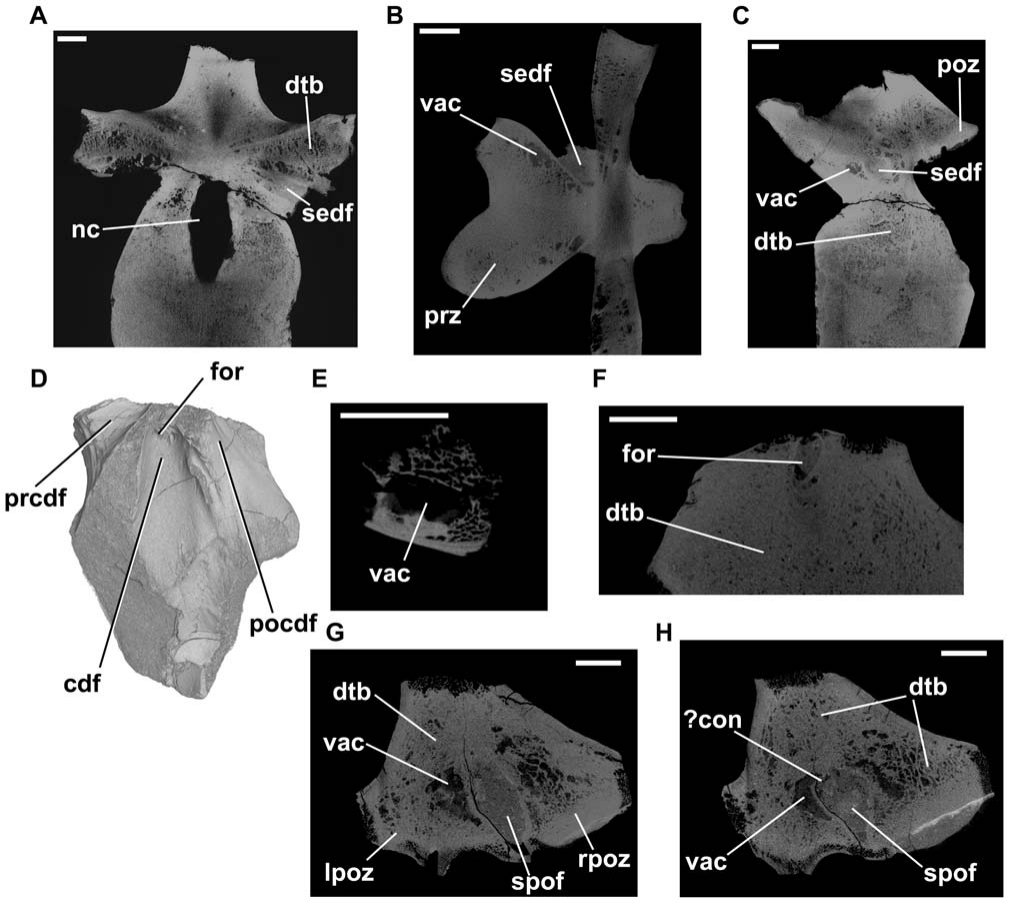
*Erythrosuchus africanus*, vertebrae and vertebral fragments. NHMUK R3592, CT cross-sections (only the neural arch and the dorsal part of the centrum were scanned): A: transverse section, taken at a point level to the anterior margin of the transverse process. B: axial section through neural arch at a point level with bases of postzygapophyses. C: parasagittal section taken at point just lateral to right border of neural canal. D: left lateral view of NHMUK R3592 ‘fragment A’ (CT rendering). E: axial section of ‘fragment A’, illustrating cavity present within the neural arch. F: parasagittal section of ‘fragment A’, illustrating sediment-filled canal that runs through bone dorsomedially from the deepest part of the infradiapophyseal fossa. G: transverse section through ‘fragment B’, illustrating vacuity within left postzygapophysis. H: transverse section through ‘fragment B’, illustrating possible connection between vacuity within left postzygapophysis and the postspinal fossa. Abbreviations: cdf, centrodiapophyseal fossa; ?con, possible connection between postspinal fossa and vacuity; dtb, dense trabecular bone; for, foramen; lpoz, left postzygapophysis; nc, neural canal; pocdf, postzygapophyseal centrodiapophyseal fossa; poz, postzygapophysis; prcdf, prezygapophyseal centrodiapophyseal fossa; prz, prezygapophysis; rpoz, right postzygapophysis; sedf, sediment-infilled external fossa; spof, spinopostzygapophyseal fossa; vac, larger intertrabecular vacuities within bone. All scale bars equal 10 mm.

**Figure 7:**
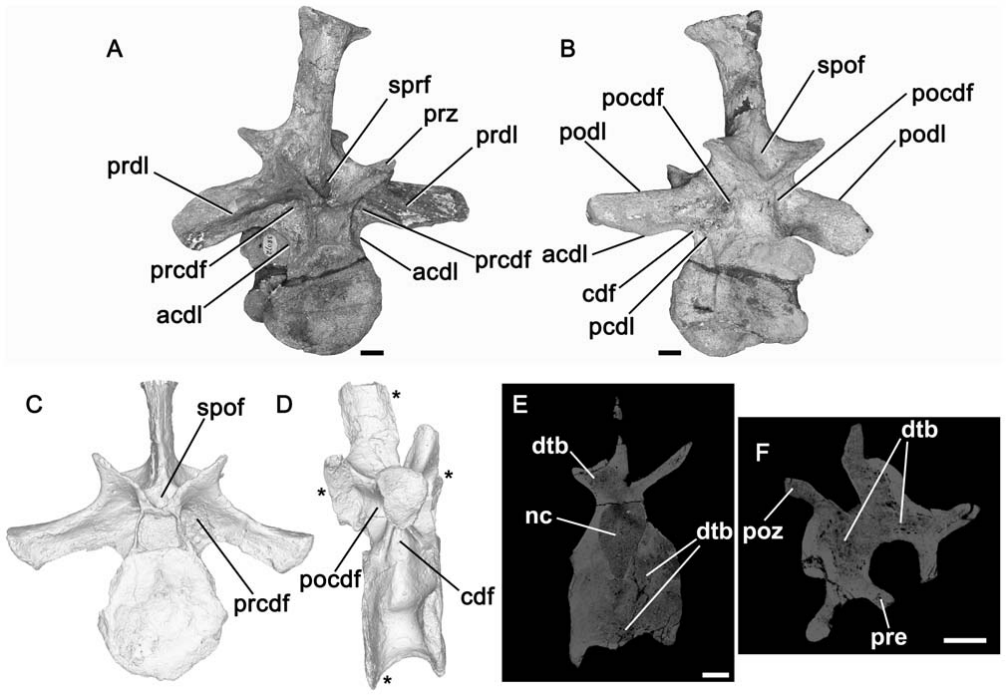
Phytosauria indet., vertebrae. A, B: NHMUK OR38072, dorsal vertebra in anterior (A) and posterior (B) views (photographs). C-F: SMNS unnumbered, dorsal vertebra in anterior (C) and right lateral (D) views with sections through the specimen (E, F). Abbreviations: acdl, anterior centrodiapophyseal lamina; cdf, centrodiapophyseal fossa; dtb, dense trabecular bone; nc, neural canal; pcdl, posterior centrodiapophyseal lamina; pocdf, postzygapophyseal centrodiapophyseal fossa; podl, postzygodiapophyseal lamina; poz, postzygapophysis; prcdf, prezygapophyseal centrodiapophyseal fossa; prdl, prezygodiapophyseal lamina; prz, prezygapophysis; spof, spinopostzygapophyseal fossa; sprf, spinosprezygapophyseal fossa. All scale bars equal 10 mm.

Proterosuchid vertebrae have not been described in substantial detail, and possible pneumaticity in this group has not been discussed previously. The presacral vertebrae of *Chasmatosaurus yuani* ( =  *Proterosuchus*) appear to lack foramina or well-developed neural arch fossae/laminae, with the exception of a weakly developed web-like PPDL (Young [76]:figs. 6, 7; Young [77]: fig. 1; Charig and Sues [70]: fig. 5). In general the strongly developed neural arch fossae and laminae of *Erythrosuchus* and many crown archosaurs seem to be absent in proterosuchids.

#### Archosauriformes: *Erythrosuchus africanus*

NHMUK R533, R3592, R8667, dorsal vertebrae. References: Gower [34], [79].

NHMUK R8667 is a series of five articulated mid–posterior dorsal vertebrae, numbered consecutively beginning with the most anterior (see Gower [34]:fig. 2). Vertebrae 2–4 are relatively complete, lacking only the diapophyses and neural spines. Vertebra 1 is relatively incomplete, with only the posterior third of the centrum and neural arch preserved. Vertebra 5 is represented by the anterior half of the centrum and most of the neural arch including the left diapophysis, although the right diapophysis, postzygapophyses, and neural spine are missing. Proximal rib fragments partially obscure the left lateral surfaces of the centra and neural arches of vertebrae 2, 4 and 5. In general, cross-sections through diapophyses, centra, neural arches and spines indicate that the bony interiors of the vertebrae are comprised mostly of dense trabecular bone [7]. However, in most cases, cross-sections are not available at the level of the foramina that pierce the neural arch.

The centra are spool-shaped with strongly pinched lateral surfaces. A fossa occurs on the dorsal third of the centrum, ventral to the neurocentral suture. This fossa is deepest on the right lateral surface of vertebra 2, and is shallower in vertebrae 3 and 4. The fossae appear to be generally shallower on the left side when compared to the right. The margins of the fossae are not defined by abrupt breaks-in-slope, clear ridges or lips of bone, nor are foramina visible within the fossae, and so they cannot be distinguished from the blind fossae that are common on the lateral surfaces of the centra of archosauriforms [29].

Well-defined laminae (PCDL, PPDL, PRDL, PRPL) occur on the neural arches, and define deep neural arch fossae. The deepest part of the centrodiapophyseal fossa is obscured in most cases by either sediment or overlying rib fragments, but is well exposed on the left side of vertebra 3. In the most dorsomedial part of the fossa is a cluster of three foramina of different sizes separated from one another by paper-thin bony septae. These three foramina are all infilled with sediment. At least four, and possibly five or more, additional small foramina are present within the centrodiapophyseal fossa. Because this fossa is not adequately exposed on other vertebrae or on the other side of vertebra 3, variation in the number, size and placement of foramina is unknown.

The prezygapophyseal centrodiapophyseal fossa is large in vertebrae 2 and 3, but decreases in size posteriorly. Numerous large infilled foramina occupy the deepest part of the prezygapophyseal centrodiapophyseal fossa, and there is strong variation in the number and shapes of foramina both along the column, with the number and size of foramina generally decreasing posteriorly, and on either side of individual vertebrae. Indeed, all vertebrae that can be examined (2–5) have strongly asymmetrical left/right patterns of foramina. The largest foramina within each prezygapophyseal centrodiapophyseal fossa reach up to 12 mm in diameter and are separated from adjacent foramina by thin bony septae. In some cases, these large foramina appear to be composed of conjoined smaller openings. For example, the largest opening on the right side of vertebra 2 has a maximum width of 12 mm, and is clearly formed of at least five conjoined openings, two of which remain partially separated by a thin bony septum that projects into the opening. The bony septum separating the large medial and lateral openings is only 1 mm thick at its thinnest point and is itself pierced by a very small foramen. The septae that separate the foramina have a surface texture that is very distinct from that of the surrounding cortical bone, with a strongly pitted and less ‘finished’ appearance.

The postzygapophyseal centrodiapophyseal fossa is not exposed in any of the vertebrae. Dorsal to the transverse process is a cluster of foramina at the base of the neural spine; these foramina are not set within distinct fossae. As elsewhere on the neural arch, adjacent foramina are separated from one another by thin bony septae and there are strong left/right asymmetries in the number and size of foramina. Unlike other parts of the neural arch, the size/number of foramina do not clearly decrease posteriorly.

Although cross-sections reveal that the majority of the neural arch and centrum is composed of dense trabecular bone, there are some substantial paired vacuities in the neural arch, just dorsal to the level of the transverse process, as noted by Gower ([34]:fig. 2C). In vertebra 5, these vacuities have a regular, smoothly rounded, oval outline, and are about 10 mm deep and 3–4 mm in transverse width. The anteroposterior extent of these vacuities is unknown. It is not clear if these vacuities had any connection to the exterior of the bone.

CT slices for NHMUK R8667 and NHMUK R533 ([29]:fig. 6) reveal little of their internal structure, probably due to the large size and high density of the specimens. CT data for an anterior dorsal vertebra of NHMUK R3592 (see Gower [79]) are of higher fidelity, and reveal some details of the internal trabeculae (Fig. 6A–C). In general, the interior of the element appears to be composed of densely packed trabecular bone. The bone density is quite heterogeneous, with larger intertrabecular spaces (reaching up to about 8 mm in diameter) concentrated within the neural arch, lateral to the neural canal. However, there are no clear connections between these larger spaces and external fossae/foramina, and at least some of the external foramina (e.g., those positioned dorsal to the transverse process) open into areas of dense and apparently apneumatic bone.

Several neural arch fragments from NHMUK R8667 were also scanned. One of these (arbitrarily referred to as “NHMUK R8667, fragment A”) is a left neural arch pedicel, broken at the level of the transverse process (Fig. 6D–F). The centrodiapophyseal fossa is well preserved; its deepest part narrows to a narrow canal with an elliptical outline (approximately 7 mm by 2.5 mm wide). This canal extends dorsomedially, is infilled with dark sediment, and is clearly visible in CT cross-sections. Unfortunately, because the specimen is broken at the level of the transverse process, it is not possible to determine whether it connected to internal chambers. Two smaller foramina positioned posterior to this canal extend only a very short distance into the bone and do not connect to large internal chambers. The dorsal breakage of the neural arch fragment exposes a cross-section through the arch immediately ventral to the transverse process. Although most of the cross-section is composed of dense trabecular bone, a large oval cavity is present within the neural arch medial to the prezygapophyseal centrodiapophyseal fossa. This cavity has well-defined and regular walls (Fig. 6E), and is infilled with black sediment. Unfortunately, because of the breakage of the neural arch the total dimensions cannot be determined, or whether this cavity is connected to an external foramen.

CT data for other neural arch fragments also indicate that the majority of the arch is composed of dense trabecular bone, but that there is a high degree of heterogeneity in the size of the intertrabecular spaces. For example, in “NHMUK R8667, fragment B” a large cavity (reaching up to 19 mm in its maximum dimension) occurs within the left postzygapophysis adjacent to the spinopostzygapophyseal fossa (Fig. 6G, H). This cavity and the sediment-infilled spinopostzygapophyseal fossa are possibly connected (Fig. 6H), although this is difficult to confirm from available CT data.

#### Archosauriformes: *Euparkeria capensis*

CAMZM T692. Reference: Ewer [80].

Ewer [80] noted a thin ridge of bone connecting the parapophysis and diapophysis in the dorsal vertebrae of *Euparkeria*: this corresponds to the PPDL. A small and shallow pocket-like centrodiapophyseal fossa occurs beneath the PPDL: the posterior margin of this fossa is formed by a very low anteroventral-to-posterodorsal trending ridge (CAMZM T692). A weakly developed ridge extends between the diapophysis and the prezygapophysis in an equivalent position to the PRDL. A fossa is present dorsal to the base of the transverse process in the mid-dorsals; it is not clear whether this fossa is blind or not. These fossae and laminae are typically not as well developed as those of *Erythrosuchus* and many crown archosaurs. Foramina are not generally evident in *Euparkeria*, with the exception of small nutrient openings on the lateral surfaces of some of the centra. Cervical vertebrae generally lack any development of laminae/fossae ([80]; CAMZM T692). The morphologies of the cervical and dorsal vertebrae of the euparkeriid *Osmolskina czatkowicensis* appear to be very similar to those of *Euparkeria* [81].

#### Archosauriformes: Phytosauria

Specimens: NHMUK OR38072, SMNS unnumbered, dorsal vertebrae.

Vertebrae of three phytosaur genera (listed as *Leptosuchus*, *Nicrosaurus*, and *Rutiodon*) were examined by O'Connor [7] as part of his review of PSP in archosaurs. O'Connor ([7]:fig. 13C) noted the presence of blind neural arch fossae on phytosaur vertebrae which he considered similar to the non-pneumatic fossae found in extant crocodylians that house adipose deposits. O'Connor ([7]:fig. 13C) figured NHMUK OR38072, a dorsal vertebra of a phytosaur (listed on the NHMUK catalogue as *Nicrosaurus kapffi*, although this taxonomic assignment cannot be confirmed at present). This element (Fig. 7A, B) has well-developed laminae (ACDL, PCDL, PODL, PRDL) and deep prezygapophyseal centrodiapophyseal, prezygapophyseal centrodiapophyseal, and centrodiapophyseal fossae, as well as small spinoprezygapophyseal and spinopostzygapophyseal fossae. Poor preservation means that it is not possible to determine the presence/absence of foramina within these fossae. O'Connor [7] additionally noted that cross-sections through phytosaur vertebrae demonstrated their probable non-pneumatic nature. This is confirmed by CT data for an unnumbered vertebra from the SMNS collection (also listed on the SMNS catalogue as *Nicrosaurus kapffi*, although this taxonomic assignment also cannot be confirmed) that is very similar in external morphology to NHMUK OR38072 (Fig. 7C–F). CT data indicates that the centrum and neural arch are composed of dense trabecular bone with no evidence for large internal vacuities (Fig. 7E, F).

#### Archosauriformes: Proterochampsidae, *Doswellia kaltenbachi*, *Vancleavea campi*

References: Romer [82], Arcucci [83], Dilkes and Sues [51], Nesbitt et al. [52].

Information on the morphology of the vertebrae of the enigmatic South American clade Proterochampsidae is scarce [82], [83]. There does not appear to be any significant development of laminae/fossae or foramina in the cervical and dorsal vertebrae (e.g., Romer [82]:fig. 1; Arcucci [83]:fig. 4). Similarly, the cervical and dorsal vertebrae of *Doswellia* and *Vancleavea* appear to lack well-developed laminae/fossae and foramina [51], [52].

#### Pseudosuchia: Aetosauria

NHMUK OR38070, anterior dorsal vertebra. References: Parker [84].

PSP has never been proposed for any aetosaur. Vertebral laminae and corresponding neural arch fossae are well-developed in aetosaurs and are very similar to those seen in other archosaurs. For example, the dorsal vertebrae have multiple well-developed laminae (ACDL, PCDL, PODL, PRDL, SPOL, SPRL) that define the boundaries of centrodiapophyseal, prezygapophyseal centrodiapophyseal, postzygapophyseal centrodiapophyseal, spinoprezygapophyseal and spinopostzygapophyseal fossae [84]. Parker [84] proposed that these laminae functioned in weight reduction. The presence of neural arch laminae and fossae in aetosaurs was used by Wedel [29] to support the observation that neural arch laminae and fossae in archosaurs do not provide compelling evidence for PSP. Foramina have not been previously described within the neural arch fossae in any aetosaur.

**Figure 8:**
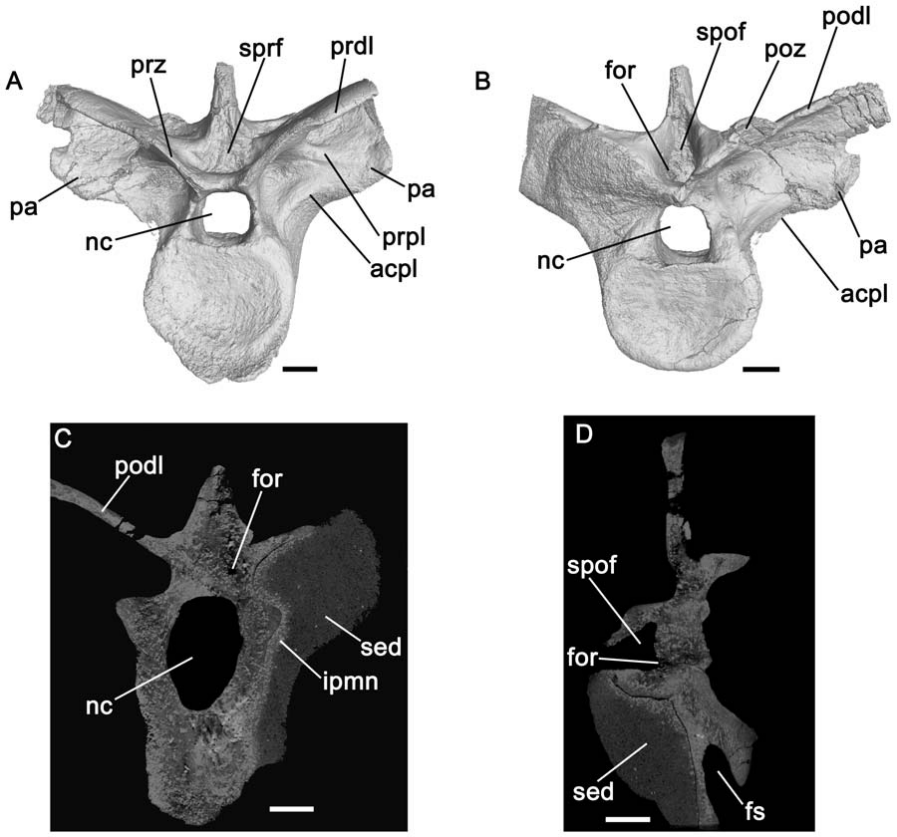
Paratypothoracine aetosaur, anterior dorsal vertebra (NHMUK OR38070). A, B: dorsal vertebra in anterior (A) and posterior (B) views. Note that there is a large volume of sediment adhering to the posteroventral surface of the left transverse process. The left transverse process was incompletely scanned and so is artificially truncated at a point just distal to the parapophysis. C: CT slice showing transverse section (in anterior view) immediately anterior to the base of the postspinal fossa. The left of the two foramina within the postspinal fossa is visible, and is surrounded by dense trabecular bone. D: CT slice showing section through the neural canal. The position of the left foramen within the postspinal fossa is marked. Note that in both CT slices intertrabecular spaces are mineral-infilled; pore spaces in the sediment immediately adjacent to the external surface of the bone are also infilled. Abbreviations: acpl, anterior centroparapophyseal lamina; for, foramen; ipmn, mineral-infilled pore spaces within sediment adjacent to the bone; nc, neural canal; pa, parapophysis; podl, postzygodiapophyseal lamina; poz, postzygapophysis; prdl, prezygadiapophyseal lamina; prpl, low ridge forming incipient prezygaparapophyseal lamina; prz, prezygapophysis; sed, sediment; spof, spinopostzygapophyseal fossa; sprf, spinosprezygapophyseal fossa. All scale bars equal 10 mm.

NHMUK OR38070 (Fig. 8) is an anterior dorsal vertebra referable to a paratypothoracine aetosaur (SJ Nesbitt, WG Parker pers. comm.). This specimen possesses relatively well-developed laminae (ACPL, PODL, PPDL, PRDL) and associated fossae. The lateral surfaces of the centrum are strongly pinched relative to the articular faces. A pair of foramina (Fig. 8) in the base of the spinopostzygapophyseal fossa are separated from each other by a broad midline septum, and similar foramina also appear to occur in the spinoprezygapophyseal fossa (although this is difficult to confirm due to imperfect preservation). Foramina cannot be identified elsewhere on the neural arch and centrum. Mineral infilling of intratrabecular spaces partially obscures details of the internal anatomy in CT slices. However, it is clear that the internal structure, including areas immediately adjacent to the foramina within the spinopostzygapophyseal fossa, comprises densely packed trabecular bone (Fig. 8C, D), which is relatively homogenous throughout the vertebra. There is no evidence for the presence of internal pneumatic cavities.

#### Pseudosuchia: Poposauroidea: *Bromsgroveia walkeri*

BIRUG 2473, dorsal vertebra. References: Galton and Walker [85], Benton and Gower [35].

**Figure 9:**
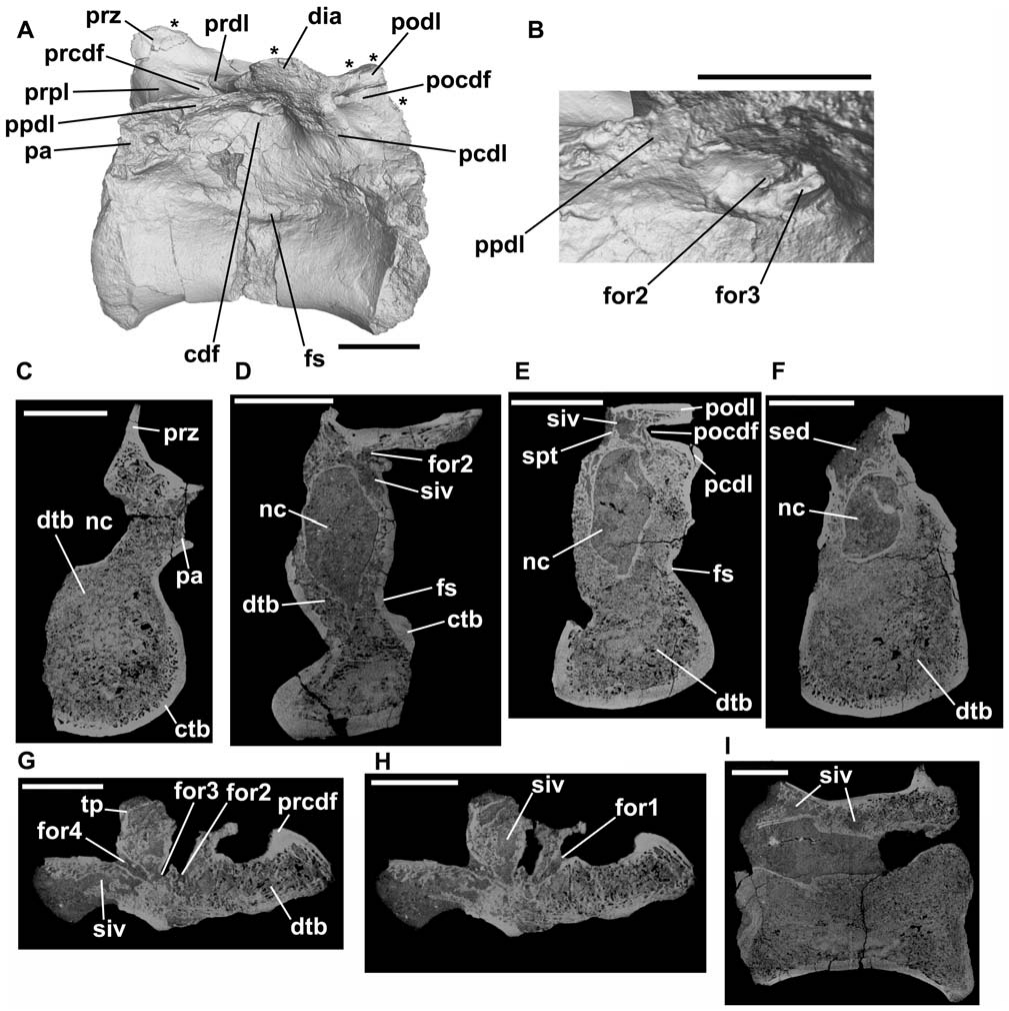
*Bromsgroveia*, dorsal vertebra (BIRUG 2473). A: left lateral view. Note that the asterisks positioned along the dorsal margin of the element correspond to positions of transverse sections shown in C–F, while the asterisk positioned adjacent to the posterior margin shows the approximate position of axial sections G and H. B: close-up of deepest part of centrodiapophyseal fossa in left lateral view showing the positions of ‘foramen 2’ and ‘foramen 3’. C: transverse section through the element close to its anterior end. Note that the centrum, neural arch pedicel, and prezygapophysis are composed of dense trabecular bone. D: transverse section through the element close to mid-length. Note the presence of a relatively large sediment-infilled intertrabecular space (siv) in the neural arch. E: transverse section through the element close to the posterior end. Relatively large paired sediment-filled intertrabecular spaces are present in the neural arch dorsal to the neural canal and are separated from one another by a bony midline septum. F: tranverse section through the element close to the posterior end. G: axial section. Note that ‘foramen 4’ extends into a sediment-infilled canal that is connected to ‘foramen 3’. H: axial section, positioned slightly dorsal to the section shown in G. ‘Foramen 1’ also extends into a sediment-infilled canal that is connected to ‘foramen 2’. I: sagittal section (anterior end of the specimen is towards the right). Two relatively large sediment-infilled intertrabecular spaces (siv) are visible in the neural arch – the posterior space corresponds to that shown in E, and the anterior space corresponds to that shown in D. Abbreviations: cdf, centrodiapophyseal fossa; ctb, cortical bone; dia, diapophysis; dtb, dense trabecular bone; for1, for2, for3, for4, foramina; fs, fossa; nc, neural canal; pa, parapophysis; pcdl, posterior centrodiapophyseal lamina; pocdf, postzygapophyseal centrodiapophyseal fossa; podl, postzygodiapophyseal lamina; poz, postzygapophysis; ppdl, paradiapophyseal lamina; prcdf, prezygapophyseal centrodiapophyseal fossa; prdl, prezygodiapophyseal lamina; prpl, prezygaparapohyseal lamina; prz, prezygapophysis; sed, sediment; siv, sediment-infilled vacuity; spt, bony septum. All scale bars equal 10 mm with the exception of B, which is equal to 5 mm.

BIRUG 2473 is a dorsal vertebra that was described by Galton and Walker [85] and Benton and Gower [35] and referred to *Bromsgroveia* (Fig. 9A). The vertebra is best preserved on the left side: the postzygapophyses, right prezygapophysis, distal left diapophysis, neural spine and right neural arch are missing. The centrum is elongate and low, with strongly pinched lateral surfaces; elongate, deep, and blind fossae are present immediately ventral to the inferred position of the (indistinguishably fused) neurocentral suture. Well-developed laminae (PCDL, PPDL, PODL, PRDL, PRPL) define the margins of three prominent fossae. The prezygapophyseal centrodiapophyseal fossa is small, shallow and narrows to a dorsoventrally compressed slit-like foramen (referred to here as ‘foramen 1’) between the PPDL and PRDL. The largest and deepest of the neural arch fossae is the centrodiapophyseal fossa, which at its deepest part contains a subfossa that is demarcated ventrally and posteriorly by low ridges (Fig. 9B). Within this subfossa two foramina are separated by a bony septum; these foramina are both elliptical with their long axes aligned in an anteroventral-to-posterodorsal direction (referred to here as ‘foramina 2 and 3’; see Figure 9B). The postzygapophyseal centrodiapophyseal fossa is larger than the prezygapophyseal centrodiapophyseal fossa and also has a foramen (‘foramen 4’) in its deepest part. There is no significant fossa or clear evidence of foramina dorsal to the transverse process (contra [35]). The fossae with foramina in their bases were interpreted as potentially pneumatic by Benton and Gower [35].

The internal morphology of this specimen is clearly visible in CT cross-sections. A layer of cortical bone surrounds the edge of the vertebra and is thickest around the margins of the centrum at the midpoint of its length but thins towards anterior and posterior ends of the centrum, and on the external surface of the neural arch. A very thin layer of cortical bone lines the neural canal. Internal to the cortical bone, most of the space is taken up by densely packed trabecular bone (Fig. 9C). The intertrabecular spacing is highly heterogenous. The bone is packed most densely within the centrum, prezygapophysis, and neural arch pedicles. By contrast, considerably larger interconnected sediment-infilled spaces occupy the transverse process and the neural arch dorsal to the neural canal, primarily within the posterior half of the vertebra. The largest and most notable of these internal spaces are at the posterior end of the vertebra. At the posterior end, much of the neural arch above the canal has broken away, and cross-sections show sediment lying above the neural canal (Fig. 9F). More anteriorly, the sediment extends into paired openings above the neural canal, separated by a very thin midline bony septum (Fig. 9E, I). These openings are approximately 2–2.5 mm in diameter, but occupy most of the transverse width of the neural arch at this point. They are connected to surrounding smaller sediment-infilled intertrabecular spaces. Foramen 4 leads into a sediment-infilled canal (Fig. 9G, H) approximately 1 mm in diameter, which connects to the intertrabecular spaces above the neural canal already described, as well as to additional sediment-infilled intertrabecular spaces within the neural arch and transverse process. Moreover, this canal connects via a sediment-infilled intertrabecular space to foramen 3 within the centrodiapophyseal fossa (Fig. 9G). Foramen 3 also has sediment-infilled connections to intertrabecular spaces within the transverse process and arch and to foramen 2. Foramen 2 is connected via a sediment infilled canal to foramen 1 within the prezygapophyseal centrodiapophyseal fossa (Fig. 9H); this canal is approximately 1 mm in width. A relatively large sediment-infilled intertrabecular space occurs within the neural arch pedicel medial and ventral to foramina 2 and 3 (Fig. 9D, I). The neural arch anterior to the point of foramen 1 does not appear to have possessed large sediment-infilled intertrabecular spaces and is composed of dense trabecular bone (Fig. 9C).

#### Pseudosuchia: Poposauroidea: *Effigia okeeffeae*

AMNH FR 30587, cervical and dorsal vertebrae. References: Nesbitt and Norell [39], Nesbitt [86].

Nesbitt and Norell ([39]:1047) noted the presence of “true pleurocoels” on the posterior half of the lateral surface of the centrum of the anterior cervical vertebrae of *Effigia*. This statement was subsequently cited as evidence of PSP in this taxon [24], [40]. Nesbitt ([86]:35) noted the similarities of these “pleurocoels” to those seen in coelophysoid theropods, but noted that: “AMNH FR 30589 bears pleurocoel-like depressions on the posterolateral portion of the centrum. The pleurocoel-like feature is a fossa with a distinct rim of bone surrounding it, which complies with Britt's definition of a true pleurocoel. However, the distinct rim of bone does not enclose a pocket, so the presence of a true pleurocoel is ambiguous”. The “pleurocoels” of *Effigia* are therefore blind fossae and do not communicate with internal vacuities; they are thus ambiguous indicators of the presence of PSP [7]. Nesbitt [49] noted that the referral of this cervical vertebra to *Effigia* was likely but not certain. Nesbitt [86] additionally noted the presence of well-developed vertebral laminae (PCDL, PODL, PPDL, PRDL) in the posterior cervicals of *Effigia*, with associated fossae.

**Figure 10:**
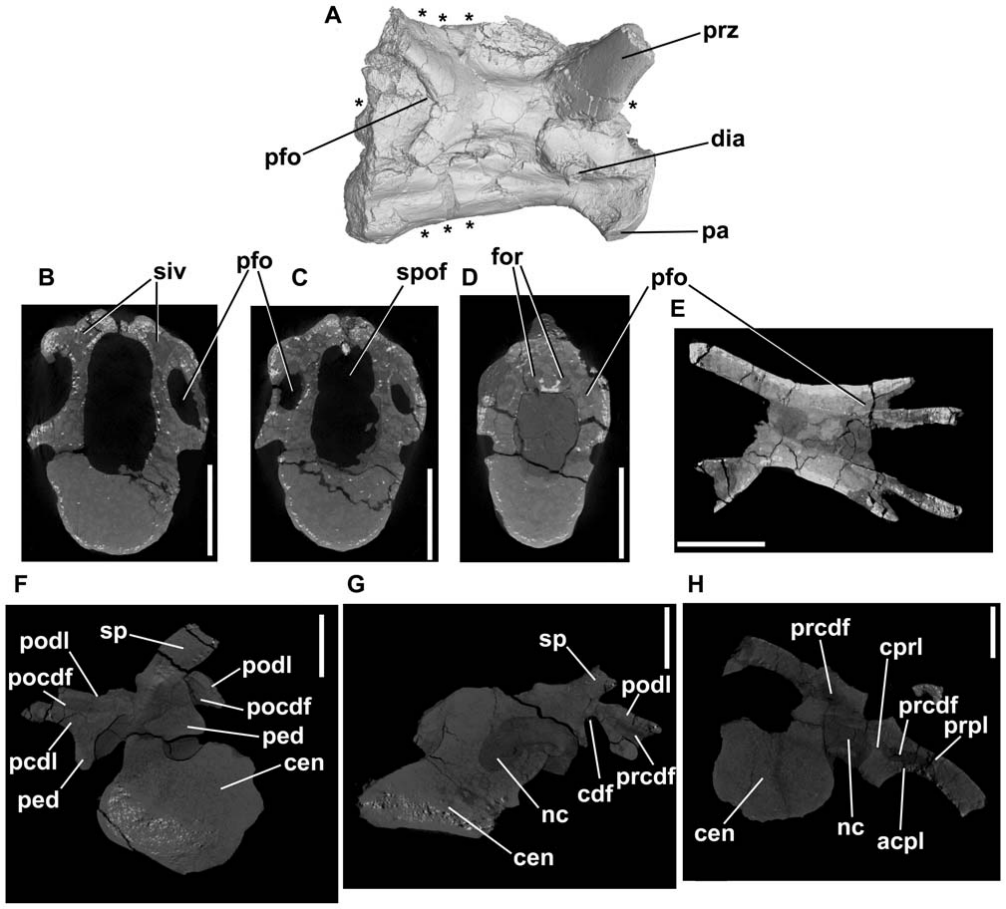
*Effigia okeeffeae*, cervical and dorsal vertebrae (AMNH FR 30587). A–E, anterior cervical vertebra in right lateral view (A) and cross-section (B–E). Asterisks dorsal and ventral to the vertebra in A indicate the positions of the transverse sections shown in B–D. Asterisks to left and right of the vertebra in A indicate the position of the axial section shown in E. F–H: CT slices showing transverse sections through AMNH FR 30587, four semi-articulated dorsal vertebrae. F: section through third preserved dorsal, immediately posterior to transverse process. G: section through third preserved dorsal, immediately posterior to transverse process. H: section through second preserved dorsal, immediately anterior to most anterior extent of neural spine. Note that in both vertebrae figured the neural arch and centrum are disarticulated. Abbreviations: acpl, anterior centroparapophyseal lamina; cdf, centrodiapophyseal fossa; cen, centrum; cprl, centroprezygapophyseal lamina; dia, diapophysis; for, foramina within base of spinopostzygapophyseal fossa; nc, neural canal; pa, parapophysis; pcdl, posterior centrodiapophyseal lamina; ped, neural arch peduncle; pfo, deep fossa on posterior of neural arch; pocdf, postzygapophyseal centrodiapophyseal fossa; podl, postzygodiapophyseal lamina; prcdf, prezygapophyseal centrodiapophyseal fossa; prpl, prezygoparapophyseal lamina; prz, prezygopophysis; siv, sediment-infilled vacuity within neural arch; sp, neural spine; spof, spinopostzygapophyseal fossa. All scale bars equal 10 mm.

Nesbitt ([86]:fig. 28C, df) figured, but did not describe, a deep fossa on the posterolateral surface of the neural arch of the anterior cervical vertebra of AMNH FR 30587 (Fig. 10A–E). This vertebra also possesses small spinoprezygapophyseal and large spinopostzygapophyseal fossae (although the ventral margin of the spinopostzygapophyseal fossa is broken). The fossa on the posterolateral surface of the neural arch has an oval outline in transverse cross-section and tapers in dorsoventral height and transverse width anteriorly, extending to a point just posterior to the mid-length of the vertebra (Fig. 10B–E). Anterior to this point the internal structure of the vertebra is hard to determine in CT data; however, the intertrabecular spaces appear to be relatively larger within the neural arch than in the centrum. Relatively large (maximum dimensions approximately 3.5 mm), sediment-infilled cavities are visible within the neural arch medial and dorsal to the fossae (Fig. 10B), at the base of the postzygapophyses. The cavities have irregular outlines, and there are no clear connections between them and the fossae. CT data also reveal that the spinopostzygapophyseal fossa divides in its deepest part into paired subfossae, similar to those seen in the aetosaur specimen discussed above. These subfossae also lack clear connections to the internal cavities.

Well-developed laminae (ACPL, PCDL, PODL, PRPL) occur in the four semi-articulated dorsal vertebrae described by Nesbitt ([86]:fig. 30) and scanned by us (Fig. 10F–H). There is no well-developed PPDL because the diapophysis and parapophysis are effectively confluent. A CPRL and a weakly developed SPRL are also evident on each side. Several well-developed fossae are present: a deep triangular prezygapophyseal centrodiapophyseal fossa; an exceptionally deep, laterally-placed centrodiapophyseal fossa with an oval pit-like depression in its deepest part; a groove-like postzygapophyseal centrodiapophyseal fossa on the posterior margin of the transverse process; a shallow spinoprezygapophyseal fossa; and a vestigial centroprezygapophyseal fossa laterally bordering the neural canal anteriorly. Nesbitt [86] reported a fossa on the base of the dorsal surface of the transverse process. However, this fossa is extremely subtle and does not resemble the condition seen in *Erythrosuchus* (see above) and *Hypselorhachis* (see below). It is not possible to determine from the external morphology whether foramina are present in the bases of the fossae. The vertebrae are taphonomically distorted, with the neural arches displaced from their articulations with the centra. Deep, longitudinally oriented fossae are also present on the lateral surfaces of the centra.

Although the neural arch fossae of the dorsal vertebrae are exceptionally deep, the neural arch pedicles, transverse processes, and neural spines appear to be solidly constructed from dense trabecular bone; there is no evidence from CT data for the presence of foramina in the fossae that open into large internal chambers (Fig. 10F–H).

#### Pseudosuchia: Poposauroidea: *Hypselorhachis mirabilis*

NHMUK R16586, anterior dorsal vertebra. Reference: Butler et al. [36].

*Hypselorhachis* is based upon a single anterior dorsal vertebra (NHMUK R16586; Butler et al. [36]:figs. 1–3) which has exceptionally well-developed laminae (PCDL, PPDL, PRDL, PRPL, SPRL, and unnamed accessory laminae) and associated fossae (prezygapophyseal centrodiapophyseal, postzygapophyseal centrodiapophyseal, centrodiapophyseal, spinoprezygapophyseal, and spinopostzygapophyseal fossae, an additional spinodiapophyseal fossa on the base of the dorsal surface of the transverse process), as well as possible foramina within the centrodiapophyseal fossa. Images based upon the CT data are poorly resolved; however, the internal morphology consists of relatively dense trabecular bone and evidence for large internal cavities is absent [36]. Butler et al. [36] concluded that there is no unambiguous evidence for PSP in this taxon.

#### Pseudosuchia: Poposauroidea: *Shuvosaurus* ( =  ‘*Chatterjeea*’) *inexpectatus*

References: Chatterjee [87], Long and Murry [88].

The cervical vertebrae of *Shuvosaurus* were not described in detail by Long & Murry [88]. However, Chatterjee ([89]:fig. 12, 1–8) figured the vertebrae (as *Postosuchus*) and described them briefly. Deep elongate fossae occupy the lateral surfaces of the centra ([89]:fig. 12, 3b, 4b). Alcober & Parrish ([90]:555) noted the presence of “distinct pleurocoels that extend most of the length of the centra just below the neural arches” and considered this morphology to be shared with *Sillosuchus*. Nesbitt ([86]:35) noted the presence of “pleurocoel-like” features in the cervical vertebrae, but did not describe or figure them in detail. Nesbitt ([48]:228) reported “pneumatic features” as a synapomorphy of *Shuvosaurus*, *Effigia* and *Sillosuchus*. The dorsal vertebrae of *Shuvosaurus* have not been described or figured adequately.

#### Pseudosuchia: Poposauroidea: *Sillosuchus longicervix*

Reference: Alcober and Parrish [90].

The holotype (PVSJ 85) of *S. longicervix* was described by Alcober and Parrish [90] and includes five partial cervical vertebrae and the last four dorsal vertebrae. The lateral surfaces of the cervicals have deep excavations that are elongated anteroposteriorly. The dorsal vertebrae were figured but not described, but also have clearly demarcated, deep excavations on the lateral surfaces of their centra and at least some well-developed neural arch laminae (although it is not clear exactly which laminae were present). No foramina can be identified in the figures provided by Alcober and Parrish [90], nor were foramina mentioned in their text. Deep fossae and well-developed neural arch laminae also appear to occur on the lateral surfaces of the anterior caudals but were not described. The cervical excavations were described as “distinct pleurocoels that extend most of the length of the centra just below the neural arches” ([90]:555). Nesbitt ([48]:30) described the cervical and dorsal excavations in *Sillosuchus* as “deep pockets (pneumatic recesses)”.

#### Pseudosuchia: Loricata: *Batrachotomus kupferzellensis*

SMNS, numerous specimens, cervical and dorsal vertebrae. Reference: Gower and Schoch [37].

Two specimens were scanned. A cervical vertebra (SMNS 80291) has ACDL, PCDL, PRDL and PODL laminae, with accompanying prezygapophyseal centrodiapophyseal, postzygapophyseal centrodiapophyseal, and centrodiapophyseal fossae, as well as a spinopostzygapophyseal fossa. A deep fossa is also present on the lateral surface of the centrum, the base of which is formed by a distinct lip of bone. Foramina are not evident in any of these fossae. Unfortunately, CT data for this specimen are poorly resolved due to minimal contrast between bone and matrix, and few details of the internal anatomy are evident. Therefore, it is uncertain if any of these fossae connected with large internal spaces.

An anterior dorsal vertebra (SMNS 80306) has very well-developed fossae and laminae. These include the PCDL, PPDL, PRDL, PRPL, and PODL, with well-developed prezygapophyseal centrodiapophyseal, postzygapophyseal centrodiapophyseal, and centrodiapophyseal fossae fossae. In addition, there is a large and deep spinodiapophyseal fossa dorsally, at the junction between the transverse process and the neural spine, a deep elliptical fossa situated on the lateral surface of the centrum, a small centroprezygapophyseal fossa positioned between the PRPL and the neural canal, and a very deep spinopostzygapophyseal fossa. There is no clear evidence for foramina within any of these fossae. The internal morphology of the specimen is generally unclear in the CT data, due to poor contrast between bone and matrix. However, it can be determined that the majority of the vertebra is made up of relatively dense trabecular bone, although there is some variation in the size of the intertrabecular spaces. There is no evidence in the CT data that any of the fossae have connections to large internal cavities.

#### Ornithodira: excluding *Silesaurus*, Pterosauria and Dinosauria

References: Romer [91], Sereno and Arcucci [92], [93].

Several Triassic ornithodirans have been described that cannot be assigned to either Dinosauria or Pterosauria; these include *Scleromochlus* (generally considered either to lie outside almost all ornithodirans or to be the sister taxon of Pterosauria [44]), and the dinosauromorphs Lagerpetidae (including *Lagerpeton* and *Dromomeron* spp.: [57], [92]), *Marasuchus* [93], and Silesauridae (including *Eucoelophysis*, *Lewisuchus*, *Pseudolagosuchus*, *Sacisaurus*, *Silesaurus* and *Technosaurus*: [58], [59], [91], [94]–[99]. There have been very few explicit statements or discussion of the presence/absence of pneumatic or potentially pneumatic features in these taxa, and available data on axial morphology is generally rather limited.

The complete presacral column of a referred specimen (PVL 3870) of *Marasuchus* was described and partially figured by Sereno and Arcucci [93]. These authors mentioned a “hollow” positioned beneath the diapophysis on the sixth to twelfth presacral vertebrae. This “hollow” appears to be bounded anteriorly by a weak PPDL and posteriorly by a weak PCDL ([93]:58, fig. 3A) and is probably equivalent to the centrodiapophyseal fossa. A weak PRCL and shallow prezygapophyseal centrodiapophyseal fossa may also be present ([93]:fig. 3A), at least in the posteriormost figured vertebra. No foramina were described. Britt ([15]:70) and Wedel [29], [100] have suggested that *Marasuchus* lacks unequivocal evidence of pneumaticity, and Wilson [25] suggested that this taxon lacked vertebral laminae.

The holotype of *Lewisuchus* includes a series of 17 presacral vertebrae [91]; this material has been only described briefly, and may be synonymous with *Pseudolagosuchus* [58]. Romer ([91]:fig. 6) described well-developed fossae on the lateral surfaces of the centra in presacral vertebrae from the posterior end of the cervical series and anterior end of the dorsal series. Moreover, he noted the presence of an anterior lamina between the diapophysis and the centrum (possibly a PPDL, although the position of the parapophysis is unclear in the published figures), and a PCDL, PRDL, and PODL. Prezygapophyseal centrodiapophyseal, postzygapophyseal centrodiapophyseal, and centrodiapophyseal fossae are clearly present ([91]:fig. 6). No foramina were described.

*Eucoelophysis* was initially described as a coelophysoid theropod [96], but has since been demonstrated to represent a non-dinosaurian dinosauriform [97], [99]. The holotype includes several dorsal vertebrae [96], but these have never been figured or described in detail. The centrum of each dorsal vertebra was described as possessing “a large, distinct, non-invasive pleurocoel on each side” ([96]:83). As discussed above, the term ‘pleurocoel’ has not been applied consistently; in this case it has apparently been used to denote the presence of a fossa (of unspecified form and depth) on the lateral surface of the centrum. Non-invasive fossae are commonly found on the lateral surfaces of archosaur centra and are not necessarily pneumatic [7].

Vertebral material for *Asilisaurus* has only been briefly described thus far [58], and pneumaticity was not discussed, but anterior cervical and sacral vertebrae lack pneumatic foramina and deep fossae.

The presence or absence of pneumaticity cannot be assessed adequately for *Scleromochlus* because of its small size and mode of preservation (natural moulds: [44]). Axial material is unknown for *Dromomeron* [57]. Only the atlantal intercentrum and caudal vertebrae are known for *Sacisaurus* [98], whereas only a few posterior dorsals, sacrals, and anterior caudals are known for *Lagerpeton* [92] and *Pseudolagosuchus* [95]; the latter have not been described or figured in detail. A single dorsal vertebra is known for *Technosaurus* [94], [99], but has not been described or figured in sufficient detail to merit discussion herein.

#### Ornithodira: *Silesaurus opolensis*

ZPAL, numerous specimens (e.g. ZPAL Ab III 404/4, 411/7, 423/1, 1299), cervical and dorsal vertebrae. References: Dzik [59], Piechowski and Dzik [101].

**Figure 11:**
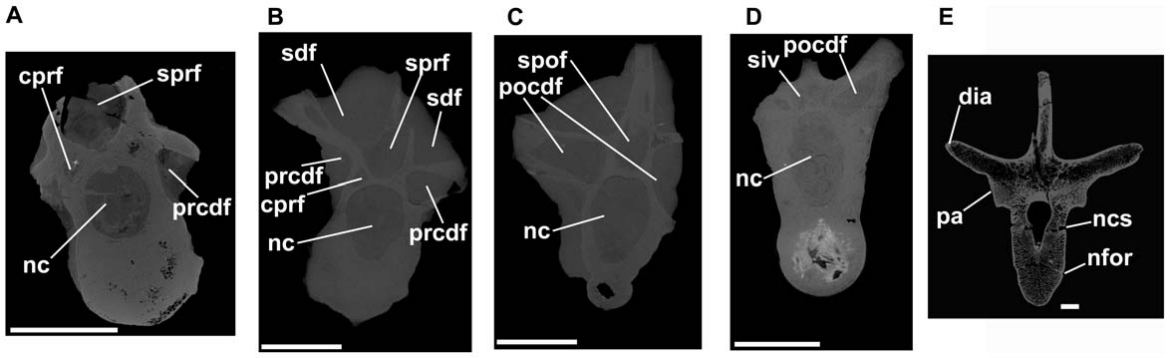
Dinosauromorpha, cervical and dorsal vertebrae. *Silesaurus* (A–D) and *Scelidosaurus* (E). A: ZPAL Ab III 1299, anterior cervical vertebra, tranverse CT section close to anterior end of specimen. B: ZPAL Ab III 423/6, posterior cervical vertebra, transverse section close to anterior end of specimen. C: ZPAL Ab III 423/6, posterior cervical vertebra, transverse section close to posterior end of specimen. D: ZPAL Ab III 404/4, posterior dorsal vertebra, transverse section close to posterior end of specimen. E: NHMUK R1111, anterior dorsal vertebra, transverse section through element close to midlength. Note that there is substantial heterogeneity in the distribution of trabecular bone, but there is no evidence of large pneumatic vacuities. Abbreviations: cprf, centroprezygapophyseal fossa; dia, diapophysis; nc, neural canal; ncs, neurocentral suture (unfused); nfor, nutrient foramen on lateral surface of centrum; pa, parapophysis; pocdf, postzygapophyseal centrodiapophyseal fossa; prcdf, prezygapophyseal centrodiapophyseal fossa; sdf, spinodiapophyseal fossa; siv, sediment-infilled vacuity within neural arch; spof, spinopostzygapophyseal fossa; sprf, spinosprezygapophyseal fossa. All scale bars equal 10 mm.

Dzik [59] and Piechowski and Dzik [101] noted that the cervical vertebrae of *Silesaurus* possess prominent laminae and fossae (referred to by Dzik as “chonoses”) but that there is no unambiguous evidence of pneumatization. Dzik [59] also noted that the fossae decrease in size posteriorly along the vertebral column. The anterior cervicals of *Silesaurus* (e.g., ZPAL Ab III 411/7, probably represents cervical 4, Piechowski and Dzik [101]:fig. 3; ZPAL Ab III 1299, Fig. 9A) do possess a complex of well-developed laminae (e.g., CPOL, CPRL, PCDL, PODL, PPDL, PRDL, SPOL, SPRL, TPOL, TPRL) that radiate from the diapophysis, including laminae that are not generally present in non-saurischian archosaurs (e.g, CPOL, CPRL, TPOL, TPRL). Numerous deep fossae are present, including prezygapophyseal centrodiapophyseal, postzygapophyseal centrodiapophyseal, and centrodiapophyseal fossae, a fossa that covers the entire lateral surface of the neural spine dorsal to the diapophysis (the spinodiapophyseal fossa), large spinoprezygapophyseal (not shown in the reconstructions presented by Dzik [59]) and spinopostzygapophyseal fossae, and a fossa positioned between the CPRL, TPRL, and neural canal (centroprezygapophyseal fossa). The spinoprezygapophyseal fossa is bisected along the midline by a transversely compressed anterior extension of the neural spine, and CT data indicate that the same is true for the spinopostzygapophyseal fossa. The spinodiapophyseal fossa is partially subdivided in ZPAL Ab III 411/7 by a subtle and weak ridge that extends posteriorly from the SPRL towards the deepest part of the fossa. Other surface irregularities also occur within the spinodiapophyseal fossa of ZPAL Ab III 411/7, including low, anteroposteriorly extending ridges in its posterior half. The centrodiapophyseal fossa is also partially subdivided at its base by a vertically oriented, rounded ridge. The neural arch fossae appear to be blind; obvious large foramina are absent. The lateral surface of the centrum is strongly pinched and depressed and is covered by the ventral extension of the centrodiapophyseal fossa, the ventral margin of which is formed by a distinct elongate ridge that extends between the parapophysis and the posterior end of the centrum. The bony laminae and fossae are so well developed that they effectively reduce the neural arch to a series of interconnected thin bony sheets. Although the development of fossae is strong, CT sections do not show any clear evidence for internal vacuities within the cervical vertebrae (Fig. 11A).

Posterior cervical vertebrae (e.g., ZPAL Ab III 423/1) have a similar pattern of fossae and laminae to the anterior cervicals, but the vertebrae are proportionately shorter and taller, with reduced spinoprezygapophyseal and spinopostzygapophyseal fossae and less strongly constricted centra. CT data do not provide evidence for internal vacuities in the posterior cervicals (Fig. 11B, C). There are prominent laminae (PCDL, PODL, PPDL, PRDL, PRPL) and fossae (prezygapophyseal centrodiapophyseal, postzygapophyseal centrodiapophyseal, and centrodiapophyseal fossae) in the dorsal vertebrae. Spinoprezygapophyseal and spinopostzygapophyseal fossae are missing in middle to posterior dorsals, as are the spinodiapophyseal and centroprezygapophyseal fossae. There are no major fossae or foramina on the centra, although very small nutrient foramina are common. In general, the fossa and laminae are less strongly developed in the dorsal vertebrae than the cervical vertebrae, and the relative sizes of the fossae decrease posteriorly along the column. ZPAL Ab III 404/4 is a large posterior dorsal vertebra lacking the postzygapophyses, most of the prezygapophyses, neural spine, and the left diapophysis and parapophysis. Breakage of the neural spine has resulted in a vertical section through the neural arch at a point level with the posterior margin of the diapophysis. In this region, most of the neural arch dorsal to the neural canal appears to be largely hollow and infilled with yellow sediment. This vacuity in the neural arch shows up in CT sections (Fig. 11D) and is similar to that seen in *Bromsgroveia* (Fig. 9E) and a specimen of *Alligator* (Fig. 3); in all cases the vacuity shows no clear connection to the exterior. With this exception, there is no evidence from CT data for large internal vacuities in the dorsal series.

#### Ornithodira: Pterosauria

References: Britt [15], Bonde and Christiansen [32], Claessens et al. [11], Butler et al. [33].

There has been relatively little detailed discussion of the distribution of PSP among pterosaurs, despite the fact that PSP has been recognised in pterosaurs for more than a century (e.g. references in Britt [15] and Bonde and Christiansen [32]). The most comprehensive review is that of Britt [15], who noted that little information is available on PSP in ‘rhamphorhynchoids’ but provided detailed descriptions of pneumatic foramina in *Dsungaripterus*, *Anhanguera*, *Pteranodon*, and azhdarchids, concluding ([15]:101) that “most, if not all, pterosaurs bore pneumatic bones”. Bonde and Christiansen [32] provided a description of the pneumatic features of *Rhamphorhynchus* and suggested that in general the large-bodied pterodactyloids have axial skeletons that are more extensively pneumatised than the smaller and phylogenetically less deeply nested ‘rhamphorhynchoids’. Moreover, they suggested that PSP is most extensive in the cervical region of many taxa, including *Rhamphorhynchus*. On the basis of examination of pterosaur material from Triassic deposits in northern Italy (see below), Bonde and Christiansen [32] concluded that early pterosaurs appear to lack PSP.

O'Connor [7] briefly discussed the presence of PSP in pterosaurs, noting in particular the high degree of appendicular pneumaticity, including pneumatisation of the distal elements of the forelimb. Claessens et al. [11] provided an overview of the distribution of PSP within pterosaurs, recognising it to be present in all major lineages of pterodactyloids and in two ‘rhamphorhynchoids’. They provided an extensive discussion of the structure of the pulmonary apparatus in pterosaurs and possible ventilatory mechanisms.

Most recently, Butler et al. [33] documented the presence of PSP in the cervical and anterior dorsal vertebrae of several of the earliest known pterosaurs, from the Late Triassic and earliest Jurassic. They pointed out that PSP is thus present in nearly all known pterosaurs and very likely the plesiomorphic condition for known members of the group.

#### Ornithodira: Dinosauria: Theropoda

References: Britt [15], Novas [102], Sereno and Novas [103], Bittancourt and Kellner [104], Nesbitt et al. [38], Martinez et al. [63], Benson et al. [12].

Several Triassic taxa, including *Herrerasaurus ischigualastensis*, *Staurikosaurus pricei*, *Chindesaurus bryansmalli*, *Eoraptor lunensis*, *Eodromaeus murphi* and *Tawa hallae* lie outside a clade comprising almost all theropods, either as proximal outgroups to Neotheropoda [38], [62] or as non-eusaurischian saurischians (outside of the clade Sauropodomorpha + Theropoda) [56], [57], although *Eoraptor* has recently been proposed to be an early sauropodomorph [63]. *Herrerasaurus*, *Staurikosaurus*, and possibly *Chindesaurus*, are generally considered to form a clade, Herrerasauridae [56].

Well-developed fossae and foramina appear to be absent from the cervical vertebrae of the herrerasaurids *Herrerasaurus* and *Staurikosaurus*, but the dorsal vertebrae possess well-developed laminae (ACDL, PCDL, PODL, PPDL, and PRDL) that frame deep prezygapophyseal centrodiapophyseal, postzygapophyseal centrodiapophyseal, and centrodiapophyseal fossae [15], [102]–[104]. However, no putative pneumatic foramina have ever been described for *Herrerasaurus* or *Staurikosaurus*, and the neural arch fossae appear to be blind. No information on the internal morphology of the vertebrae is available. Britt [15] suggested that the well-developed neural arch laminae might indicate the presence of pneumatic diverticulae: however, as discussed above, the presence of such fossae cannot be considered unambiguous evidence of PSP unless associated with foramina and large internal cavities [7], [29].

The axial column of *Eoraptor* is largely undescribed: however, Sereno et al. [24] noted that the presacral vertebrae lacked pneumatic cavities. Sereno et al. ([24]:14) mentioned the presence of “true pleurocoels” in the mid cervical vertebrae of a “new basal theropod close to *Eoraptor*”, *Eodromaeus*, which was subsequently described as possessing “pleurocoels” in posterior cervicals that open into a lateral groove that is present in other vertebrae [63].

Nesbitt et al. [38] described *Tawa* as the sister taxon to Neotheropoda, and noted the presence of “anterior pneumatic pleurocoels (as rimmed fossae)” in cervical vertebrae of this taxon, suggesting that this was evidence of postcranial skeletal pneumaticity and that the origin of cervical air sacs predates the origin of Neotheropoda. They additionally mentioned (but did not describe) the presence of anterior cervical “pleurocoels” in the herrerasaurid *Chindesaurus*, although such potentially pneumatic features were not mentioned by previous descriptive accounts [88], [99].

The presence of PSP in Neotheropoda is well established [6], [7], [12], [15], [23], [24] and the distribution of pneumatic features within this clade was discussed by Britt ([15]:table 5) and Benson et al. [12]. PSP appears to have been near universally present within Neotheropoda [6], [12], [15], and among Triassic theropods has been extensively documented in *Coelophysis bauri* [15], [105], and also reported for *Liliensternus liliensterni* ([6]:Supplementary Table 1), although in the latter case the evidence appears to be ambiguous [12]. Unambiguous PSP is limited to the cervical vertebrae in the former taxon.

#### Ornithodira: Dinosauria: Sauropodomorpha

Specimens: SMNS numerous specimens, *Plateosaurus*, cervical and dorsal vertebrae; NHMUK RU P24, *Pantydraco caducus*, partial skeleton including cervical and dorsal vertebrae. References: Britt [15], Yates [106], Wedel [29], Yates et al. [31].

As in Theropoda, the presence of PSP in derived sauropodomorphs (Eusauropoda) is well established [9], [15], [22], [26], [27], [29], [30]. Early sauropodomorphs (referred to as ‘prosauropods’ hereafter) have generally been considered to lack unequivocal evidence of PSP [15], although Britt [22] suggested that the weak fossae on ‘prosauropod’ neural arches were pneumatic in origin. Wedel [29] reviewed evidence for PSP in ‘prosauropods’ and noted that although the neural arches of ‘prosauropod’ presacral vertebrae typically possess laminae (including the ACDL, PCDL, PODL, PPDL, and PRDL) that frame deep prezygapophyseal centrodiapophyseal, postzygapophyseal centrodiapophyseal, and centrodiapophyseal fossae, the fossae themselves are generally blind. These blind fossae and laminae do not, therefore, provide unambiguous evidence for PSP. With only a few exceptions, pneumatic foramina have not been previously documented in ‘prosauropods’ [29], [31]. Wedel [29], [100] focused in detail on the report of “pleurocoel-like pits” in the Triassic early sauropodomorph *Pantydraco caducus* ([106]:14); these ‘pits’ are small fossae delimited by sharp edges on the lateral surfaces of the centra of cervical vertebrae 6–8 of the holotype (NHMUK RU P24). Wedel [100] identified these fossae as pneumatic based upon their position within the posterior region of the cervical column and the presence of distinct margins. By contrast, Wedel [29] cautioned that neither of these lines of evidence unambiguously diagnosed PSP. In conclusion, Wedel [29] concluded that compelling evidence of PSP is absent in ‘prosauropods’, with the possible exception of *Pantydraco caducus*. A more recently described taxon, *Panphagia protos*, hypothesised to represent the earliest sauropodomorph, apparently lacks evidence of PSP in its posterior cervical vertebrae [107], as does *Eoraptor*, which Martinez et al. [63] recently proposed to be a non-sauropod sauropodomorph (see above). Yates et al. [31] documented evidence of postcranial pneumaticity in a range of non-sauropod sauropodomorphs that span the ‘prosauropod’ to sauropod transition, including the early sauropod *Antetonitrus*.

**Figure 12:**
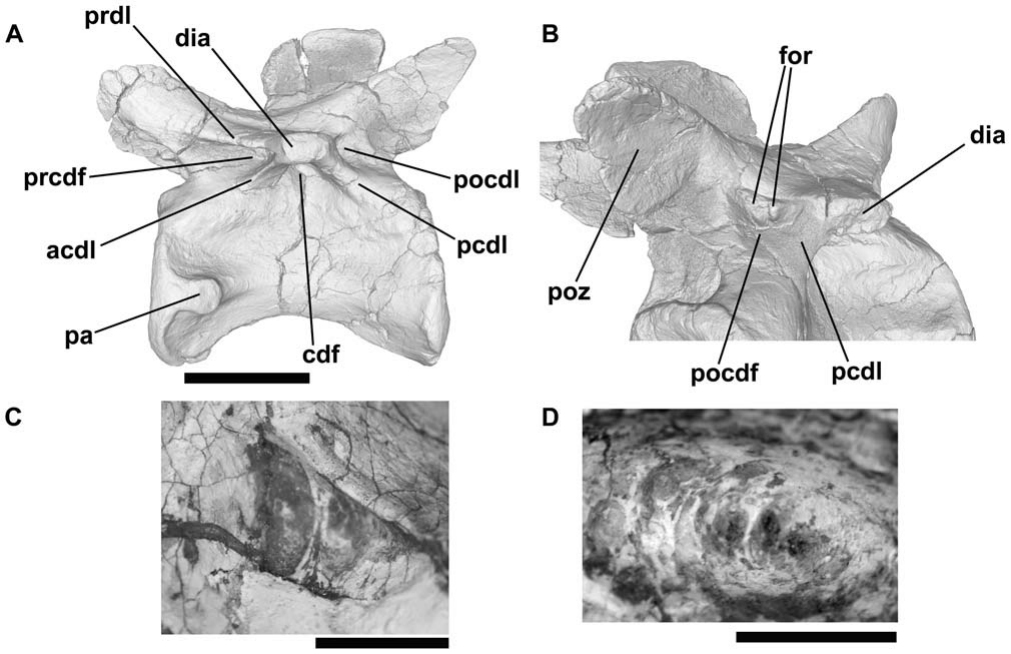
*Plateosaurus*, cervical and dorsal vertebrae. A–C: SMNS F65, cervical vertebra, left lateral view (A, CT rendering) and close-up of the right postzygapophyseal centrodiapophyseal fossa in posterolateral view showing foramina (B, CT rendering; C). D: SMNS 12950, mid dorsal vertebra, close-up of right prezygapophyseal centrodiapophyseal fossa showing cluster of foramina. Abbreviations: acdl, anterior centrodiapophyseal lamina; cdf, centrodiapophyseal fossa; dia, diapophysis; for, foramina; pa, parapophysis; pcdl, posterior centrodiapophyseal lamina; pocdf, postzygapophyseal centrodiapophyseal fossa; poz, postzygapophysis; prcdf, prezygapophyseal centrodiapophyseal fossa; prdl, prezygodiapophyseal lamina. Scale bars equal 50 mm (A) and 10 mm (C, D).

Our re-examination of material of the Triassic early sauropodomorph *Plateosaurus* indicates that the neural arch fossae of early sauropodomorphs are not necessarily blind (*contra* [29]; see also [31]). In many cases it is impossible to determine whether or not foramina are present within the fossae, due to infilling with sediment and/or poor bone surface preservation, while in other cases the neural arch fossae do indeed appear to be blind. However, in one middle dorsal vertebra (SMNS 12950) there are well-developed laminae and fossae and the prezygapophyseal centrodiapophyseal fossae have a clear cluster of foramina in their bases, separated from one another by thin bony septa (Fig. 12D). This is superficially similar to features seen in *Erythrosuchus* and ornithischian dinosaurs (see below). In one posterior cervical vertebra (SMNS F65), the postzygapophyseal centrodiapophyseal fossa (margins defined by the PCDL and a very weak PODL) is shallow and is subdivided (on both sides) by dorsoventrally extending bony septa (Fig. 12A–C). On either side of the septum are large foramina (reaching maximum dimensions of approximately 1 cm) infilled with sediment. CT scan data for this specimen are unfortunately of low quality due to poor contrast between bone and matrix, and do not reveal whether or not these foramina connect to internal chambers. Yates et al. [31] also noted a small fossa in a posterior cervical vertebra of *Plateosaurus*, subdivided by a lamina, but suggested that other specimens of this taxon lack evidence of pneumaticity.

#### Ornithodira: Dinosauria: Ornithischia

Specimens: NHMUK R1111, *Scelidosaurus harrisonii*, cervical and dorsal vertebrae; NHMUK R11521, *Mantellisaurus atherfieldensis*, dorsal vertebrae.

Ornithischian dinosaurs are usually considered to completely lack evidence of PSP ([15]:106; [22]; [29]:218). However, though previously undescribed, neural arch laminae, fossae, and associated foramina similar to those seen in *Erythrosuchus* are known in a number of ornithischians. *Scelidosaurus harrisonii* is an early thyreophoran ornithischian known from multiple well-preserved specimens from the Lower Jurassic of England. The lectotype, NHMUK R1111, is a relatively complete skeleton that has been acid-prepared completely free of matrix, and includes well-preserved cervical and dorsal vertebrae. Sections through broken vertebrae show densely packed trabecular architecture with no evidence for large internal chambers.

We scanned an anterior dorsal vertebra (labelled “D3”) that is well-preserved with the exception of a fracture in the base of the neural spine (Fig. 9D). There is minimal infilling of intertrabecular spaces. Cortical bone is missing in a few areas (on the anterior and posterior surfaces of the centrum and on the right diapophysis), revealing that the interior of the element in these areas is composed of dense trabecular bone. The centrum is spool-like and lacks well-developed fossae on its lateral surfaces; however, a number of small foramina are present, the largest of which is positioned approximately at the midpoint of the lateral surface. Fossae and weakly expressed laminae occur on the neural arch. The deepest of these is the postzygapophyseal centrodiapophyseal fossa. This fossa is often present (although variably developed) in ornithischians (RJB, PMB, pers. obs.; see below). In the deepest part of this fossa a number of small (< 1 mm in diameter) foramina occur, on both sides of the vertebra. Anteriorly a PRDL and an ACPL form the dorsal and posteroventral margins of a small and shallow prezygapophyseal centrodiapophyseal fossa, within which there are no foramina. Between the ACPL and PCDL the surface of the neural arch is gently depressed below the parapophysis, although a distinct centrodiapophyseal fossa is not well developed. There is a foramen present within this depression, adjacent to the anteroventral corner of the parapophysis. A small fossa is present posterior to this foramen, immediately adjacent to the posteroventral corner of the parapophysis: the foramen and the fossa are separated from each other by a low and short vertical ridge. The small fossa contains two small foramina within it on the right side; on the left side the fossa is blind. Foramina also occur elsewhere on the vertebra; there is an elongate elliptical foramen on the neurocentral suture, just posterior to midlength, and there is a prominent foramen on the ventral surface of the transverse process, dorsolateral to the parapophysis. There are no fossae or foramina on the dorsal surface of the transverse process.

At least superficially, the morphology of the dorsal vertebrae of *Scelidosaurus* shows similarities to *Erythrosuchus*; most notable is the presence of multiple foramina within the deep postzygapophyseal centrodiapophyseal fossa. CT data demonstrates that these structures in *Scelidosaurus* do not constitute unambiguous evidence of PSP. The vertebra is composed of densely packed trabecular bone surrounded by a thin layer of compact cortical bone. The density of the bone is heterogeneous within the vertebra: areas of highest density include areas of the centrum and neural arch adjacent to the neural canal, anterior and posterior ends of the centrum, the parapophyses and transverse processes, and the distal part of the neural spine. Density is lowest in the neural arch between the neural canal and the base of the neural spine. In no part of the vertebra are there notably large vacuities or chambers indicative of pneumaticity. The foramina within the deep posterior fossa open into an area of relatively dense trabecular bone, and the same is true of the foramina positioned ventral to the parapophysis and elsewhere on the vertebra.

Similar features are also known in a specimen of *Mantellisaurus atherfieldensis* (NHMUK R11521) from the Early Cretaceous of the UK. This specimen is well preserved, and includes an articulated series of three dorsal vertebrae. In general, these vertebrae lack fossae/laminae on the neural arch and centrum. However, a deep postzygapophyseal centrodiapophyseal fossa is present, and is bordered dorsally by a PODL and anteroventrally by a PCDL. Clusters of small (2–3 mm in diameter) foramina occur within the bases of these fossae, resembling the condition in *S. harrisonii* and *Erythrosuchus*. Although the specimen was not CT scanned due to its large size, broken sections through transverse processes and neural spines fail to show any unambiguous evidence for PSP.

## Discussion

### Distribution of unambiguous evidence for PSP among early archosauromorphs

Understanding the distribution and early evolution of PSP among archosaurs and closely related taxa is a highly challenging task given the ambiguous nature of most of the available anatomical evidence. Anatomical features, such as neural arch fossae, laminae, and ‘pleurocoels’, which have been historically used to recognise PSP in saurischian dinosaurs, are widespread (although generally less well developed) among Triassic archosauriforms (including non-archosaurian taxa), as demonstrated herein. However, such features are potentially associated with soft tissues other than the respiratory system [7], which has led recent workers to attempt to establish unambiguous criteria for identifying PSP, focusing on the presence of large internal cavities connected to the exterior of the element via large cortical bone foramina [7]. The focus on unambiguous evidence is conservative, however, and may result in genuinely pneumatic anatomical features being dismissed: consequently, the distribution of PSP among extinct taxa is likely to be underestimated. This is particularly problematic when considering the origins of PSP, where one might expect pneumatic features to be cryptic or less strongly expressed [30]. Moreover, even this ‘unambiguous’ criterion for recognising PSP is far from unproblematic – no definition has been given for how large a ‘large foramen’ must be before it can be recognised as pneumatic. Seemingly pneumatic foramina in birds and pterosaurs are highly variable in size (e.g. Fig. 4), and may be small features only a few millimetres in diameter. Distinguishing such small foramina in extinct taxa from nutrient foramina, which in extant taxa may connect to large internal intertrabecular spaces (e.g. Fig. 3), may not be possible.

Among archosaurs, three major clades (Pterosauria, Sauropodomorpha, Theropoda) are recognised to contain members with unambiguous evidence of PSP, all of which belong to Ornithodira. Recent work suggests that PSP is nearly universally present in known pterosaurs [11], [15], [33], although it should be noted that the earliest pterosaur specimens discovered thus far are from the middle–late Norian of the Late Triassic [108], whereas the pterosaur lineage must extend into at least the late Anisian of the Middle Triassic [48], [58], so it remains possible that early pterosaurs lacking pneumaticity will be discovered eventually.

Among theropods, PSP is widely distributed in neotheropods, although the evidence for PSP is ambiguous in some of the earliest ‘coelophysoid’ neotheropods such as *Liliensternus*. PSP has also been suggested to occur in several non-neotheropod theropods [24], [38], but this is based upon non-invasive ‘pleurocoels’ on the lateral surfaces of centra – features that provide only ambiguous evidence of PSP [7]. Nevertheless, the positional similarity and phylogenetic congruence (and continuity) of the ‘pleuocoels’ of the Triassic non-neotheropod *Tawa* to the unambiguously pneumatic fossae of later theropods [61], as well as their positional congruence with the “common pattern” of theropod pneumaticity [12], supports a pneumatic interpretation [12], [38]. Other non-neotheropod theropods (e.g., *Herrerasaurus*, *Staurikosaurus*) also lack unambiguous evidence of PSP. As a result, unambiguous evidence of PSP among theropods is limited to Neotheropoda, although it is possible that it can be extended further phylogenetically within Theropoda, at least as far as *Tawa*.

Among sauropodomorphs, unambiguous evidence of PSP has been considered to be absent in the non-sauropod ‘prosauropods’ [29] with ambiguous evidence documented for *Pantydraco* ( =  *Thecodontosaurus*). Our observation of large foramina situated within neural arch fossae in the cervical vertebrae of *Plateosaurus* suggests a need for a broad re-examination of the evidence for PSP in ‘prosauropods’ (see also [31]). It is possible that pneumatic features have been overlooked because of incomplete preparation, poor preservation, incomplete descriptions, or a lack of appreciation of their importance, particularly given that they are likely to be smaller features than in sauropods and might be restricted to just a few vertebrae in the column. Yates et al. [31] described PSP in a range of mostly non-eusauropod sauropodomorphs, further highlighting the need to reassess the early evidence for pneumaticity in this clade. At this stage, we urge sauropodomorph workers to search for and document both the presence *and* absence of possible pneumatic features in detail.

Among the remaining ornithodiran taxa, unambiguous evidence of PSP appears to be absent in all ornithischians and all non-dinosaurian dinosauromorphs. Even in *Silesaurus*, which possesses very well-developed vertebral laminae and fossae in the cervical and anterior dorsal vertebrae, unambiguously pneumatic foramina remain unknown.

Among pseudosuchian and non-archosaurian archosauriforms, we have similarly been unable to identify unambiguous evidence of PSP. Neural arch foramina are present in a number of taxa, and may be relatively large (e.g. *Erythrosuchus*), but we have been unable to find any instances in which they connect demonstrably to large internal chambers, and similar external features in other taxa (e.g. ornithischian dinosaurs) are not coincident with unambiguous evidence for PSP. In *Bromsgroveia*, we documented the presence of foramina connecting to internal chambers: however, the foramina and the internal chambers are relatively small, and similar features are observed in the apneumatic vertebrae of extant crocodilians (Fig. 3). ‘Pleurocoels’ on vertebral centra have been reported in several poposauroid pseudosuchians, but as in non-neotheropod theropods, these features provide only ambiguous evidence for PSP. We note, however, that the apparently exceptional development of the ‘pleurocoels’ of the poposauroid *Sillosuchus* [48] indicates that this taxon is worthy of further more detailed investigation.

In summary, unambiguous evidence of PSP within archosaurs is limited currently to all known pterosaurs, nearly all neotheropods (but not non-neotheropod theropods), and all eusauropods and some closely related non-eusauropod sauropodomorphs, such as *Antetonitrus*, but perhaps not in the majority of the earliest ‘prosauropod’ taxa.

### Are the inferred air sac systems of theropods, sauropodomorphs, and pterosaurs homologous?

The inferred presence of a heterogeneously partitioned pulmonary system with distinct gas exchange and ventilatory components, including anteriorly and posteriorly located air sacs, in pterosaurs, sauropodomorphs, and neotheropods, raises questions about the homology of this system in these three closely related clades. If a maximum parsimony argument is adopted, the absence of unambiguous osteological evidence for PSP in non-dinosaurian dinosauromorphs, non-neotheropod theropods, at least some early sauropodomorphs, and ornithischians would indicate that unambiguous PSP likely arose on at least three independent occasions (pterosaurs, sauropodomorphs, and neotheropods) with the corollary that unambiguous osteological evidence of PSP was probably absent in the ancestral ornithodiran ([29], [33], [109]). An alternative scenario in which PSP was present in the ancestral ornithodiran would require numerous losses and reversals (e.g. in *Marasuchus*, silesaurids, ornithischians, non-neotheropod theropods, some non sauropod sauropodomorphs).

However, although a conservative interpretation of the osteological evidence suggests at least three independent acquisitions of PSP, it is possible that the underlying soft-tissue system is in fact common to (and homologous across) all ornithodirans. It is possible that ornithodiran taxa lacking PSP possessed the same soft tissue systems, but that these did not invade the postcranial skeleton: after all, not all of the pneumatic diverticulae in extant birds invade bones – some are situated in the intersticies between other soft tissues, leaving no osteologial traces [2], [18]. Testing this hypothesis in the absence of unambiguous osteological markers is difficult, but it is noteworthy that there are striking similarities in the distribution and development of the unambiguous osteological correlates for PSP in all three clades (including the ontogeny of PSP in extant birds) that support the deep homology of the underlying soft tissues. In the earliest known pterosaurs [33], sauropodomorphs [29], and neotheropods [6], [12], [15] with unambiguous PSP, evidence for pneumaticity is limited to the postaxial cervical and anterior dorsal vertebrae and associated ribs. This matches the “common pattern” of PSP observed in extant birds [5], [8] as well as the earliest ontogenetic stages in extant birds with more extensive PSP [30]: a similar “common pattern” of cervical and anterior dorsal pneumaticity also applies almost universally to non-avian neotheropods [12]. It is striking that the ambiguous evidence of PSP (vertebral fossae and laminae) in non-dinosaurian dinosauromorphs (*Silesaurus*), non-neotheropod theropods (*Tawa*), and ‘prosauropods’ (*Pantydraco*, *Plateosaurus*) is restricted to, or most strongly expressed in, the cervical and anterior dorsal vertebral column, and this topographical and phylogenetic congruence provides support for these features being pneumatic.

The evolution of PSP also appears to be similar in each of these three clades, although more detailed analyses are required (particularly for pterosaurs and sauropodomorphs) to clarify evolutionary patterns. In phylogenetically more deeply nested taxa (i.e. taxa separated by a greater number of nodes from the root of the tree) within each clade, there is a tendency for PSP to be extended anteriorly along the vertebral column (resulting in pneumatisation of the axis and atlas) and posteriorly (resulting in pneumatisation of the posterior dorsal, sacral, and anterior caudal vertebrae). Moreover, appendicular PSP occurs in pterosaurs [11], some non-avian theropods [12], [24], and birds [7], [8]. Increases in the degree of skeletal pneumaticity beyond the common pattern present in early taxa appear to be linked to increases in body size in all three groups [8], [11], [12] with this relationship quantitatively demonstrated for non-avian theropods [12], supporting the idea that at least one important function of PSP may be mass reduction, at least in theropods [8], [9], [11], [12], [29].

The basis of modern comparative biology is to work from an initial position in which similarity is interpreted as homology. We propose that the underlying pulmonary soft-tissue system of pterosaurs, sauropodomorphs, and neotheropods should be considered homologous, and that this implies that this soft-tissue system, including anteriorly and posteriorly positioned air sacs (cervical and abdominal air sacs), was present in the ancestral ornithodiran, at some point during the Early–Middle Triassic, as well as in non-dinosaurian dinosauromorphs and (at least plesiomorphically) in ornithischian dinosaurs (see also: [29], [33], [109]).

Very large body size (reaching up to 22,000 kg according to some estimates: [110]) evolved repeatedly within Ornithischia, with the largest ornithischians exceeding the largest theropods, and many sauropods, in body mass. If the soft-tissue system unpinning the evolution of PSP was present in the ancestral ornithodiran, then the *potential* to evolve PSP should have been present in the earliest ornithischians. It is puzzling that no ornithischians possess unambiguous evidence for PSP, given their 160 million years of evolution and their close relationship with taxa that clearly possess this feature [7]. It is possible that the apparent absence of PSP in this clade might reflect either: secondary reduction or loss of air sacs in this lineage [29], [33], [109]; the presence of an anatomical or developmental constraint that prevented the evolution of PSP; the possession of pneumatic diverticula that failed to invade the skeleton; that the selection pressure for reduction of skeletal mass was insufficiently strong for PSP to evolve; or that our hypothesis of homology is incorrect. Unfortunately, current data do not permit us distinguish among these alternatives.

Our hypothesis of homology raises a key question that we discuss in greater detail below: if a heterogeneously partitioned pulmonary system including air sacs is plesiomorphic for Ornithodira, could its phylogenetic distribution extend even further within the archosaur tree and be plesiomorphic for Archosauria or an even more inclusive clade?

### When did the ornithodiran air sac system evolve?

Farmer and Sanders [111], [112] demonstrated that unidirectional airflow, previously thought to be a unique avian character among extant vertebrates, occurs in the American alligator, and regions of the alligator lung may be homologous with avian air sacs. This raises the possibility that unidirectional airflow evolved prior to the pseudosuchian/avemetatarsalian split and was inherited by all archosaurs [18], [111], [112]. The ancestral archosaur has also previously been proposed to have possessed a multi-chambered lung with partial separation of the pump and exchanger [7], [18]. As discussed above, we hypothesize that a non-exchange air sac system is plesiomorphic for (and homologous across) Ornithodira. The split of pseudosuchian and avemetatarsalian (including Ornithodira) lineages occurred before the end of the Early Triassic [48], [66], [67]. The earliest known ornithodiran body fossil is *Asilisaurus* from the late Anisian (c. 242–244 Ma) of Tanzania [58], which postdates the inferred Early Triassic origin of avemetatarsalians by 5–10 Myr. Recent phylogenetic analyses recognize numerous synapomorphies of Ornithodira, including some or all of the following: elongation of the cervical column, reduction of the forelimb, absence of osteoderms, absence of the interclavicle and clavicle, elongation of distal hindlimb elements (tibia and metatarsals), bunched metatarsus, and other modifications to the hindlimb [38], [47], [48]. Ornithodirans have often been proposed to plesiomorphically possess accelerated growth rates relative to pseudosuchians ([113], [114]), although ongoing work is questioning this idea [115], [116], and ornithodirans may possibly (although controversially) have plesiomorphically possessed filamentous integument (‘protofeathers’: [117]). Thus, although no taxa are currently known that unambiguously subtend the avemetatarsalian branch leading from the ancestral archosaur to the ancestral ornithodiran, a substantial number of morphological changes occur along this lineage.

Many of these morphological changes are linked to the inferred presence of a fully erect gait, cursoriality, and bipedality in the mostly small-bodied and slenderly built early ornithodirans, and may indicate increased activity levels and heightened metabolic rates. A plausible hypothesis, therefore, is that as proposed previously [18] the ancestral archosaur possessed lungs with unidirectional airflow and incomplete separation of the pump and exchanger (but with regions of low parenchymal density that could form the precursors of air sacs), but lacked the true air sac system of ornithodirans (with a complete separation between pump and exchanger), and that the latter evolved in concert with locomotor and other changes in the earliest avemetatarsalians during the Early to early Middle Triassic (Fig. 1). This hypothesis would be consistent with recent work suggesting that elevated evolutionary rates occurred during the earliest phase of archosaur evolution [118]. Furthermore, this hypothesis would suggest that more efficient lung ventilation initially evolved in concert with increased activity levels and (possibly) heightened metabolic rates; subsequently PSP originated and was elaborated on in multiple ornithodiran lineages independently, particularly in large-bodied and/or flying taxa [8], [11], [12].

Assuming that air sacs and respiratory diverticula are homologous for at least Ornithodira still implies that there has been substantial change (and homoplasy) in the development and extent of PSP within the constituent lineages. Several members of each of the major ornithodiran clades, not all of them merely early representatives, lack or have restricted unambiguous PSP. If members of some of these lineages had respiratory diverticula associated with only ambiguous evidence of PSP (vertebral laminae, fossae and weakly developed ‘pleurocoels’), then the obvious question is: were similar features in non-ornithodiran (and even non-archosaurian) archosauriforms also associated with respiratory diverticula, and thus indicative of incipient PSP? The case for answering this question becomes stronger if the features providing ambiguous evidence for PSP in early ornithodirans are found to be phylogenetically continuous (or continuous enough to infer homology) with those of pseudosuchians and non-archosaurian archosauromorphs. Although the inferred phylogenetic relationships of Triassic archosauriforms remain partly in a state of flux, current understanding suggests that it is plausible that some of these features (in particular, the well-developed cervical and dorsal vertebral laminae and associated fossae) may prove homologous across Triassic archosaurs (see Fig. 1).

Although O'Connor [7] and Wedel [29] have suggested that the vertebral features of *Erythrosuchus* and some other ambiguously pneumatic taxa are non-specific fossae (potentially associated with fat deposits) or vascular foramina, we have observed substantial differences between the osteological features in the vertebrae of *Erythrosuchus* and those of modern crocodilians. In particular, the fossae and laminae are much more strongly developed and prominent in the former, and the extent of their development matches the “common pattern” distribution of unambiguous PSP features in ornithodirans (concentrated in cervical and anterior dorsal vertebrae). We posit that if *Erythrosuchus* was a saurischian dinosaur, then its vertebral features would not be so readily dismissed as apneumatic, and that this is reason for caution. Similarly well-developed fossae and laminae do not appear to occur outside Archosauriformes, suggesting that something different is occurring within this clade. We do not dispute O'Connor's [7] conclusion that unambiguous evidence of PSP has a more restricted distribution than suggested by Gower [34]. However, we believe that the interpretation of the osteological axial structures among archosauromorphs has not been addressed in sufficient detail and that it would be wiser to flag the deeper origins of PSP as an unresolved issue, rather than one that has been answered definitively. Future resolution may be provided by the development of new methods (such as histology) to unambiguously identify non-invasive PSP or if unambiguous osteological indicators of PSP are identified in pseudosuchians by future discoveries, with poposauroids such as *Sillosuchus* probably representing the best candidate group for identifying such features.

### Detecting PSP in fossil archosauromorphs

In this study we applied CT scanning to the identification of PSP – did this help? In some cases CT data clearly have helped to clarify the absence of unambiguous PSP. However, several interesting specimens (including those of *Erythrosuchus*) did not scan well and revealed no new information on internal structure. It is not always possible to determine a priori whether a specimen will produce good CT data, though the majority of the poorly scanned specimens in our study were clearly heavily mineralised. In the absence of improved scan technology or methodology (e.g., packing dense specimens in a medium such as sand), the internal structure of such specimens will more likely be revealed by fortuitous breaks or more destructive examination. Thus, while CT scanning is a useful additional tool in investigations of PSP, it is not a universal solution.

## Methods

### Sampling of taxa for micro-computed tomography (µCT)

We sampled a phylogenetically broad group of early archosauriforms, focusing primarily on taxa for which PSP has previously been proposed (e.g., *Erythrosuchus*, *Bromsgroveia*, *Effigia*) or which possess morphological features that have often been considered indicative of PSP (e.g., laminae, fossae, and foramina: *Hypselorhachis*, Phytosauria, *Silesaurus*). Because Gower [34] mentioned possible indicators of PSP in the archosauromorph clade Rhynchosauria, we sampled two vertebrae referable to the Tanzanian rhynchosaur *Stenaulorhynchus*. We additionally sampled a vertebra of the early dinosaur *Scelidosaurus*, because this taxon possesses similar morphological features to those seen in *Erythrosuchus* (see below) yet belongs to a clade (Ornithischia) generally considered to possess an apneumatic skeleton. For comparative purposes and to help provide additional guiding data on interpretation of CT data we sampled material of four major lineages of extant sauropsids with known presence/absence of PSP: birds (*Anser*, *Struthio*), crocodilians (*Alligator*), squamates (*Varanus*), and chelonians (*Chelonoidis*). The specimens sampled are listed in Text S1.

Because unambiguous PSP is most commonly developed in the cervical and dorsal vertebrae in saurischian dinosaurs and extant birds [8], [12], [15], [100], and because PSP appears first in cervical vertebrae both evolutionarily and developmentally [12], [33], [100], we focused our examination on these parts of the axial skeleton. The choice of taxa examined using µCT was constrained by the presence and accessibility of axial material and specimen size. For example, some Triassic taxa lack preserved cervical and dorsal vertebrae, while material for other taxa (e.g., *Euparkeria*, *Ticinosuchus*) is preserved in articulation/association in slabs that make examination via µCT difficult or impossible. The majority of the material scanned is housed or accessioned in the collections of the Natural History Museum (NHMUK), London, although material was additionally loaned from other institutions. Where a choice of multiple cervical/dorsal vertebrae was available for a taxon, we selected vertebrae that were well preserved and that showed the greatest development of features (e.g. laminae, fossae, foramina) that might potentially be indicative of PSP. For extant squamates and chelonians we selected large specimens from large species, assuming that this would give us the best opportunity of detecting the osteological features of interest.

### Micro-computed tomography (µCT) methods

Specimens were micro-CT scanned at NHMUK between July 2008 and February 2009 by S Walsh and R Abel using a HMX-ST CT 225 System (Metris X-Tek, Tring, UK). Data were reconstructed using CT-PRO software version 2.0 (Metris X-Tek) and rendered/examined using VGStudio MAX 2.0. Micro-CT data are archived at NHMUK and the National Geoscience Data Centre (Keyworth, UK).

## Supporting Information

Text S1 **List of fossil specimens that were CT-scanned, and summary of comparative CT-data for extant sauropsid taxa.**
(DOCX)
