# Supplementary material for: Reassessment of the Evidence for Postcranial Skeletal Pneumaticity in Triassic Archosaurs, and the Early Evolution of the Avian Respiratory System
Source: PLoS One. 2012 Mar 28;7(3):e34094. doi: 10.1371/journal.pone.0034094 (PMC3314707; doi:10.1371/journal.pone.0034094)
Supplement: Text S1 — List of fossil specimens that were CT-scanned, and summary of comparative CT-data for extant sauropsid taxa. (DOCX) [file pone.0034094.s001.docx]

Supplement to:

**Reassessment of the Evidence for Postcranial Skeletal Pneumaticity in Triassic Archosaurs, and the Early Evolution of the Avian Respiratory System**

**Richard J. Butler,^1,2^^[[1]](#footnote-1)^* Paul M. Barrett,^2^ and David J. Gower^3^**

**1** GeoBio-Center, Ludwig-Maximilians-Universität München, Richard-Wagner-Straße 10, D-80333 Munich, Germany, **2** Department of Palaeontology, Natural History Museum, Cromwell Road, London, SW7 5BD, UK, **3** Department of Zoology, Natural History Museum, Cromwell Road, London, SW7 5BD, UK

**Fossil specimens used for CT-scanning**

Aetosauria: NHMUK OR 38070 (Paratypothoracisini indet.), dorsal vertebra. “Keuper” (Late Triassic; more accurate stratigraphic data not available but probably from the Löwenstein Formation), Stuttgart, Germany.

*Batrachotomus kupferzellensis*: SMNS 80291, cervical vertebra, SMNS 80306, anterior dorsal vertebra. Erfurt Formation (Middle Triassic: Ladinian), Baden-Württemberg, Germany.

*Bromsgroveia walkeri*: BIRUG 2473, dorsal vertebra. Bromsgrove Sandstone Formation (Middle Triassic: Ladinian), Worcestershire, England.

*Effigia okeeffeae*: AMNH FR 30587, cervical vertebra, four partially articulated dorsal vertebrae. “Siltstone Member”, Chinle Formation (Late Triassic: Norian–Rhaetian), New Mexico, USA.

*Erythrosuchus africanus*: NHMUK R533, R3592, R8667, dorsal vertebrae. Burgersdorp Formation, *Cynognathus* Assemblage Zone B + C (Middle Triassic: Anisian), South Africa.

*Hypselorhachis mirabilis*: NHMUK R16586, anterior dorsal vertebra. Lifua Member of the Manda Beds (Middle Triassic: latest Anisian), Tanzania.

Ornithischia: NHMUK R1111, *Scelidosaurus harrisonii*, anterior dorsal vertebra (dorsal ‘D3’), Lower Lias (Early Jurassic: Simemurian–lower Pliensbachian), Dorset, UK.

Phytosauria: SMNS unnumbered, Phytosauria indet. dorsal vertebra, Löwenstein Formation (Late Triassic: Norian), Baden-Württemberg, Germany.

Rhynchosauria: NHMUK R36618B, *Stenaulorhynchus stockleyi*, cervical and dorsal vertebrae, Manda Beds (field locality U2/26), Tanzania, Middle Triassic (latest Anisian).

Sauropodomorpha: SMNS F65, *Plateosaurus* sp., posterior cervical vertebra. Löwenstein Formation (Late Triassic: Norian), Baden-Württemberg, Germany.

*Silesaurus opolensis*: ZPAL AbIII 1299, anterior cervical vertebra; ZPAL AbIII 423/1, two posterior cervical vertebrae; ZPAL AbIII 423/6, posterior cervical vertebra; ZPAL AbIII 1294, anterior dorsal vertebra; ZPAL AbIII 404/4, posterior dorsal vertebra. All specimens from unnamed unit, Krasiejów, Poland, Late Triassic (late Carnian).

1. [↑](#footnote-ref-1)
